# Interplay between Mixed and Pure Exciton States Controls Singlet Fission in Rubrene Single Crystals

**DOI:** 10.1021/jacs.5c02993

**Published:** 2025-06-24

**Authors:** Dmitry R. Maslennikov, Marios Maimaris, Haoqing Ning, Xijia Zheng, Navendu Mondal, Vladimir V. Bruevich, Saied Md Pratik, Yifan Dong, John W. G. Tisch, Andrew J. Musser, Vitaly Podzorov, Jean-Luc Bredas, Veaceslav Coropceanu, Artem A. Bakulin

**Affiliations:** † Department of Chemistry and Centre for Processible Electronics, 4615 Imperial College London, London W120BZ, U.K.; ‡ Department of Physics and Astronomy, Rutgers University, Piscataway, New Jersey 08854, United States; § 5922 Cornell University, Department of Chemistry and Chemical Biology, Ithaca, New York 14853, United States; ∥ Department of Chemistry and Biochemistry, 8041 The University of Arizona, Tucson, Arizona 85721, United States; ⊥ Department of Physics, Imperial College London, London SW72BW, U.K.

## Abstract

Singlet fission (SF) is a multielectron process in which one singlet exciton **S** converts into a pair of separated triplet excitons **T**. SF is widely studied as it may help overcome the Shockley–Queisser efficiency limit for semiconductor photovoltaic cells. To elucidate and control the SF mechanism, great attention has been given to the identification of intermediate states in SF materials, which often appear elusive due to the complexity and fast time scales of the SF process. Here, we apply 14 fs-1 ms transient absorption techniques to high-purity rubrene single crystals to disentangle the intrinsic fission dynamics from the effects of defects and grain boundaries and to identify reliably the fission intermediates. Our data demonstrates that above-gap excitation directly generates a hybrid vibronically assisted mixture of singlet state and triplet-pair multiexciton **[S/TT]**, which rapidly (<100 fs) and coherently branches into pure singlet or triplet excitations. The relaxation of **[S/TT]** to **S** is followed by a relatively slow and temperature-activated (48 meV activation energy) incoherent fission process. The SF competing pathways and intermediates revealed here unify the observations and models presented in previous studies of SF in rubrene and offer alternative strategies for the development of SF-enhanced photovoltaic materials.

## Graphical abstract

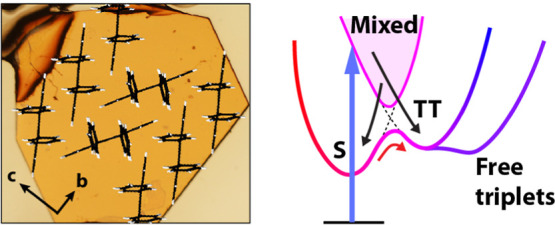

## Introduction

Coherent dynamics are identified to underpin an increasing number of ultrafast processes in molecular photochemistry, from biological light harvesting^1–3^ to donor–acceptor charge transfer^4,5^ to molecular exciton multiplication processes.^6–9^ Frequently these processes entail conversion between optically bright and dark electronic states, and the coherence manifests through the mechanistic involvement of distinct superposition states, often derived from strong coupling to vibrational modes. Such vibronic coupling induces mixing between bright and dark states, leading to formation of new quantum states possessing different extents of the properties of both original states, as can be seen, for example, in spectroscopic data.^10^ These mixed states serve as key transition states or intermediates along the potential energy landscape. This framework has been widely applied to singlet fission (SF), in which a photoexcited singlet state **S** converts into a pair of separated triplet states, mediated by a spin-entangled multiexciton state **TT**.^8,9,11–21^ The latter state exhibits key hallmarks of its mixed character—combinations of singlet-, charge-transfer-, and triplet-derived spectral signatures;^22–24^ stabilization relative to the uncoupled parent states;^11^ and ability to emit photons and diffuse over micron length scales despite being nominally dark and localized.^25–28^ Whether exothermic or endothermic, its formation on ultrafast time scales is found to be driven by strong vibronic coupling.^8,9,17,29–31^

A corollary of this mechanism is that this multiexciton character should likewise be mixed into the photoexcited **S** state, though not necessarily at the Franck–Condon point. In this case, the coherent singlet fission process could be understood as the projection of the **TT** character from the mixed state onto the photoproduct triplet multiexciton. Such direct mixing of **TT** into **S** is predicted theoretically in several systems,^9,30,32,33^ but this effect has only been inferred experimentally from the observation of coherent dynamics.^9^ In the best known example, the direct excitation of a coherently mixed **[S/TT]** state was initially proposed in pentacene and tetracene based on the presence of low-energy signatures in transient photoelectron spectroscopy.^6,7^ However, subsequent analysis^34^ and recent angle-resolved photoelectron spectroscopy^35^ indicate these systems follow the standard conversion from a pure **S** state into mixed **TT**. It thus remains unclear whether the **[S/TT]** state can be directly photoexcited, despite its established ability to emit photons.^11,22,25,26,36^

We address this question using single crystalline rubrene of very high purity. Rubrene has nearly isoenergetic **S** and **TT** states and long triplet diffusion lengths,^37–39^ making it optimal for SF applications. However, the *C*_2*h*_ symmetric stacking in rubrene results in negligible electronic coupling between **S** and **TT**,^38^ resulting in especially pronounced sensitivity to static and dynamic disorder. The dynamics of SF in rubrene have thus been controversial, with reported mechanisms ranging from <100 fs coherent to >10 ps incoherent channels.^14,40–48^ These early studies were limited by the complex interplay between intrinsic fission dynamics related to the crystal structure and extrinsic dynamics tied to defects and grain boundaries. In addition, in the leading time-resolved studies, the assignments to **S** and **TT** states were limited by the absence of distinct spectral fingerprints not overlapped with the ground-state absorption or thermal artifacts.^14,40,49^

Here, we study high-purity single crystals using sub-14 fs-to-ms transient absorption spectroscopy (TAS) in the near-infrared, a background-free region where unique fingerprints of **S**, **TT**, and a mixed **[S/TT]** state can be readily distinguished. We recover dynamics of slow, thermally activated SF from **S** similar to prior reports.^41–46^ However, our key finding is that **S** is not the initial photoexcited state. Instead, the initial species contains unique signatures of a mixed **[S/TT]** state. Thermalization causes this state to collapse either into **TT**, which leads to a fast fission channel, or into **S**, with the latter population responsible for the slow fission channel. The branching from **S/TT** and its dependence on the excitation frequency reconciles the competing claims of coherent and incoherent fission in rubrene and demonstrates that even dark, multiexciton states can be directly photoexcited through strong vibronic coupling.

## Results and Discussion

Rubrene single crystals were grown using the physical vapor transport (PVT) technique, as it is well-established to provide molecular crystals with a very low concentration of defects, thereby minimizing the influence of static disorder on charge and exciton dynamics.^26,50^ Previous work on tetracene has demonstrated that the fission rate and mechanism inferred from polycrystalline films are dominated by grain boundaries and other types of disorder and differ significantly from those in single crystals.^51,52^ Similarly, the TAS data of highly disordered samples is expected to be contaminated with signals originating from defects and grain boundaries (see semiamorphous rubrene data in Supporting Information, Section III), which may lead to data misinterpretation. To distinguish the intrinsic SF properties from extrinsic effects, it is therefore imperative to study low-defect crystalline materials. We note that the photoluminescence and other photophysical properties of organic solids, even in their highly purified single crystalline form, are very sensitive to trace impurities and structural defects,^53–57^ thus requiring special care during the material growth. Thus, for this study, we prepared high-purity PVT-grown rubrene single crystals similar to those used in the recent first demonstration of a photo-Hall effect in organic semiconductors.^58^

Figure 1a shows a photograph of a representative PVT-grown rubrene single crystal studied in this work, with the corresponding molecular packing motif schematically overlaid on the image. The crystal is a hexagonally shaped thin plate, with its largest facet corresponding to the bc crystallographic plane (the so-called high-mobility plane of orthorhombic rubrene; in some studies,^50^ an alternative “ab” notation has been used). The hexagonal habitus was used to match the crystal axis orientation with the shape of the crystal (Section II of Supporting Information). Figure 1c shows the absorption spectra of the crystal for the incident light linearly polarized along the 0C and 0B crystallographic axes. In our case, the thickness of the crystals (tens of micrometres) was significantly greater than the thickness of the photoexcited layer (Section I of Supporting Information).

**Figure 1:**
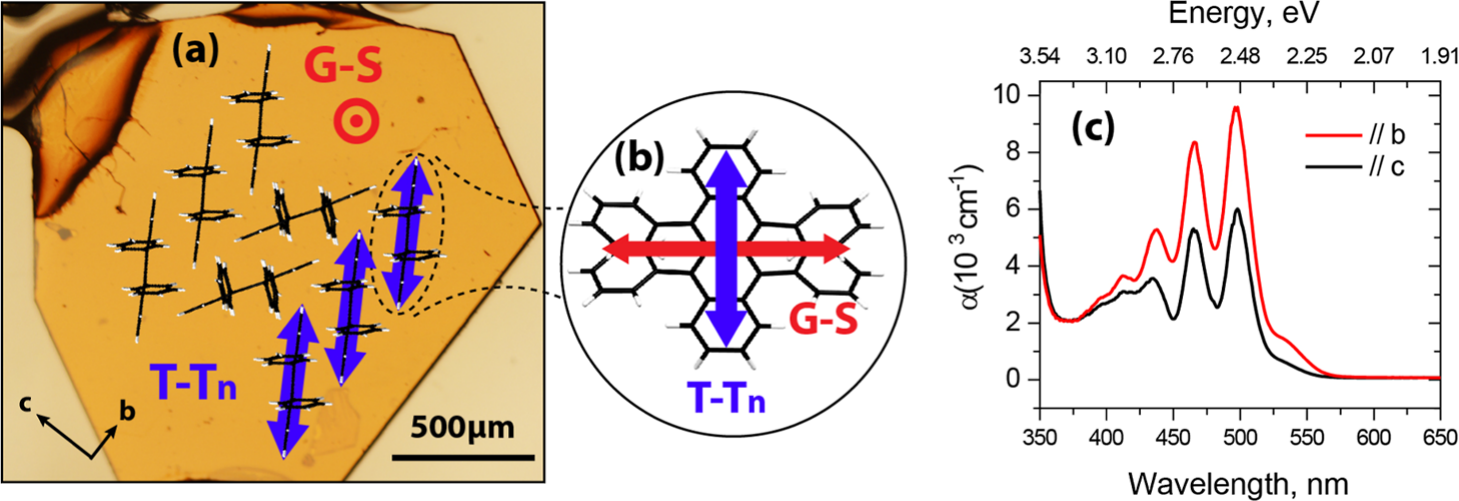
(a) Microscopy image of the bc-plane upper facet of a rubrene single crystal in correspondence with the molecular-packing schematics. (b) Illustration of a rubrene molecule with the blue [red] arrow indicating the **T** → **T**_***n***_ [**G → S**] transition dipole moment direction. (c) UV–vis absorption spectra of a rubrene single crystal for the photoexcitation polarized along *b* and *c* axes of the crystal.

We addressed exciton dynamics in the crystal using transient absorption spectroscopy (TAS) on three instruments with temporal resolutions of <14 fs, 200 fs, and 10 ns. The results were obtained using probes in the near-infrared spectral range, which is uncontaminated by ground-state absorption and thermal artifacts.^59,60^ Moreover, this range contains unique fingerprints of the key states in the SF pathway in many other acenes: narrow vibronic resonances for **T → T**_***n***_ transitions and broad bands for **S → S**_***n***_ transitions.^20,29,61–63^ In crystalline rubrene, these states can be further distinguished due to the significant anisotropy that arises from the herringbone molecular packing.^50,64^ For example, the transition dipole moments of the **G → S** and **T → T**_***n***_ transitions are orthogonal (Figure 1b).^40^ To balance the signal coming from the photoinduced absorption (PIA) of singlet and triplet excitons, the probe polarization was oriented approximately at 60° relative to the 0B crystal axis (±5° due to manual positioning of the crystal).

Figure 2a presents full TAS data from the single crystal shown in Figure 1a. The results from the three setups were scaled and merged to capture the full excited-state evolution from 14 fs to 50 μs. The probe region of sub-14 fs experiments was narrower than for other experiments due to the technical limitations of supercontinuum generation. Slight shifts of shared spectral features indicate this procedure is accurate to within 5 nm. On the ns/μs time scale we detect a single species with 13 μs lifetime, characterized by two sharp transitions at 878 and 985 nm separated by a 1300 cm^–1^ spacing. These features are widely observed in solutions and thin films of polyacene derivatives^10,23,24,29,61,65^ and the lowest-energy peak closely matches our DFT-calculated gap between relaxed **T** and **T**_***n***_ states. We thus assign these to a vibronic progression of **T → T**_***n***_ transitions of either separated free triplets **T** or electronically decoupled triplet pairs. We directly observe the formation of this state within 100 fs and the slow (>ps) part of the signal growth matches the decay of a broad photoinduced absorption beyond 1000 nm. The latter band is typical of the **S** state in numerous polyacenes^10,61–63,65^ and reveals slow SF, consistent with the incoherent SF model. We note that the use of the term “coherent” fission refers to the coherence between electronic and vibronic states, but not to the spin coherence, which cannot be probed by our spectroscopic methods, but is generally assumed to be maintained through singlet fission and has been directly observed in rubrene.

**Figure 2:**
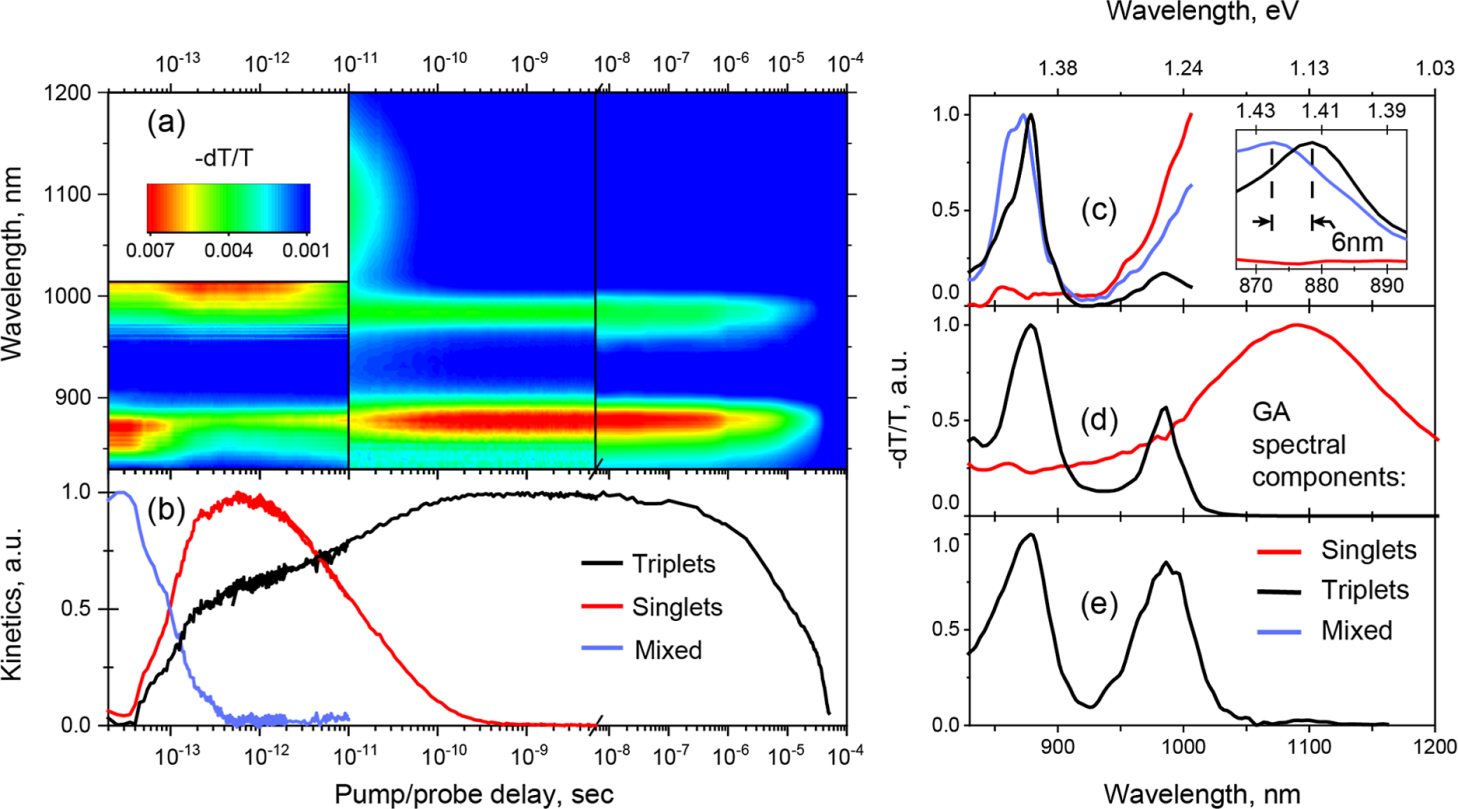
Room temperature transient absorption in a rubrene single crystal measured using three different setups (a) with temporal resolutions of <14 fs (830–1010 nm), 200 fs, and 10 ns (830–1200 nm). Kinetics (b) and spectra (c–e) of the **S**, separated triplets **T**, and **[S/TT]** states provided by the global analysis (GA) of the experimental data sets.

However, we find that **S** is not the initial photoexcited state. On the <100 fs time scale, we resolve a distinct species with a sharp band centered at 865 nm. Closer examination reveals this feature is associated with a broad photoinduced absorption >1000 nm, and its decay tracks the rise of **S**. This qualitative analysis indicates a two-step progression: from an unknown state to **S** and from **S** to free triplets **T**. To identify the initial state and disentangle its interplay with the other species, we spectrally decompose the data in each temporal range using a combination of singular value decomposition and a genetic algorithm. This method permits extraction of the characteristic spectral species without any predetermined kinetics scheme.^66^ The resulting population kinetics and spectra are presented in Figure 2b. We find good agreement between the extracted triplets **T** spectra in all ranges and the **S** spectra corroborate the analysis above. The initial species contains a mixture of characteristics of the **T** → **T**_***n***_ and **S** → **S**_***n***_ transitions. However, it cannot be described as a simple linear combination of singlet and triplet states; for instance, the 865 nm band is shifted by 6 nm relative to the separated triplets band **T**. Based on the hybrid character of this spectrum, we assign the initial species to a mixed **[S/TT]** multiexciton state—a new quantum state which is a product of **S** and **TT** interactions. This mixed state possesses properties of both **S** and **TT**, allowing ESA transitions to both **S**_***n***_ and **T**_***n***_ states.

The transient absorption features of the initially formed [**S/TT**] state include both singlet-like (**S** → **S**_***n***_) and triplet-like (**T** → T_***n***_) components, as [**S/TT**] new eigenstates are superpositions of **S**, **TT** and their vibrational excitations.^9^ This reflects its nature as a multiexciton eigenstate that retains partial electronic character from both the singlet and triplet manifolds.^10^ Due to the mixing interactions, the energies of the transitions for the **[S/TT]** can be slightly different than those for pure states leading, for example, to a 6 nm spectral shift observed for the **T** → **T**_***n***_ peak. Similar hybrid states have been identified as the immediate product state of SF in solutions and thin films^10,16,22,25,29^ and some works identify the blue-shift of photoinduced absorption peaks relative to separated triplets as a signature of the multiexciton binding energy.^11,22^ However, this is the first explicit observation that this mixed state can be directly photoexcited.

While previous studies on strongly coupled SF systems like dimers indicated that triplet pair states may have a distinct NIR signature in acene materials,^62,67^ we did not observe major modifications of the TA spectra upon state mixing. This is in agreement with other studies where mixed-state formation was distinguished not by new spectral bands, but slight shifts of the fingerprints of the parent state^16,22^ or even no direct spectral signatures at all.^15,68^ The spectral signatures of mixed states and coupled **TT** states are likely to be strongly dependent on material factors such as intermolecular coupling. In rubrene crystals, the intermolecular interactions are strongly suppressed.

The population dynamics reveal two channels of triplet formation. The photoexcited **[S/TT]** state decays with 77.0 ± 2.2 fs time constant, resulting in parallel formation of independent **S** and **TT** components. The time scale of <100 fs is typical of internal conversion and vibrational relaxation in the excited states^69,70^ and is consistent with the above-gap, vibrationally hot excitation in our 14 fs experiment. Such branching in the decay channels matches the coherent mechanism discussed above, in which the photoexcited **[S/TT]** superposition relaxes into **S** or **TT** configurations weighted by their contribution to the initial wave function.^71^ Under 400 nm excitation conditions, this coherent channel provides ∼40% of the final triplet yield (Figures 2b and 4d). Subsequently, the **S** population peaks at ∼200 fs and undergoes further SF with a time constant of 9.9 ± 0.8 ps. This slow fission rate suggests that **S** requires energy to overcome a potential energy barrier to convert back to the mixed state [**S/TT]** or directly into **TT**. That is, the relaxed **S** is stabilized relative to **TT** and does not couple to it.

We note that sub-100 fs fast conversion of the initially photoexcited **[S/TT]** to pure **S** and **TT** states leads to the creation of coherent superpositions of vibronic levels in these product states. The resulting superposition causes distinct oscillations in the TA signal in both the **S** and **TT** probe regions. We observe multiple beating frequencies, all corresponding to characteristic vibrational modes of rubrene, with the most prominent features at 75 cm^–1^ and 340 cm^–1^ (Figure S9, Section IV of Supporting Information). Based on their appearance exclusively in the PIA region and their restriction to low frequencies, we assign these to excited-state vibrational coherences. This observation highlights the ultrafast and direct nature of the photoexcitation process and is consistent with previous impulsive spectroscopy studies of rubrene.^14^ Although the excitation pulse duration (∼14 fs) could in principle generate vibrational coherences up to ∼2400 cm^–1^, we do not observe modes above 400 cm^–1^. This absence is not attributed to limited time resolution, but rather to the fact that coherence is generated in the product **S** and **TT** states as a result of the rapid (∼70–80 fs) decay of the initially formed **[S/TT]** state, rather than within the **[S/TT]** state itself. Thus, the effective bandwidth of the impulsive population transfer is limited to ∼420 cm^–1^, naturally excluding higher-frequency modes. This mechanism differs from conventional impulsive vibrational excitation, where coherence is launched directly in the initially populated excited state by a sub-20 fs pulse.^72,73^ In this context, we note that similar arguments about the interplay between formation time of the state and accessible vibrational bandwidth were discussed previously.^73^

Our control experiments (Figure S16) show that the incoherent fission dynamics are identical for different pump polarization and the effects of anisotropic fission^68^ are negligible, while the yield of coherent fission can be varied by up to 10% depending on polarization of excitation beam.

To evaluate the energetics of the slow SF channel, we performed further temperature-dependent measurements in this regime. Figure 3 shows the temperature dependence of the extracted **S** and **T** dynamics and spectra. The kinetics (Figure 3a,b) demonstrate that fission slows dramatically at low temperatures, to a time constant of 118 ± 24 ps at 125 K. This result confirms the endothermic nature of the picosecond channel of SF in rubrene; though **S** and **TT** are nearly isoenergetic,^14,38,40^ relaxed **S** faces a sufficient barrier to **[S/TT]** or **TT** to significantly impact the dynamics. Upon cooling, we further observe that the spectral signatures of **S** and **T** narrow at a similar pace (Figure 3c,d and Section V of Supporting Information). At the same time, the **S** peak undergoes a blueshift, which is not observed in the **T** peaks. The energy spacing between the **T** peaks remains constant at 1300 cm^–1^ and matches the energy of the vibrational mode most coupled to electronic states, observed in Raman measurements (Figure 3e). Following previous studies, we attribute the narrow triplet signatures to significantly different degrees of delocalization for singlet and triplet excited states in organic single crystals.^74,75^

**Figure 3:**
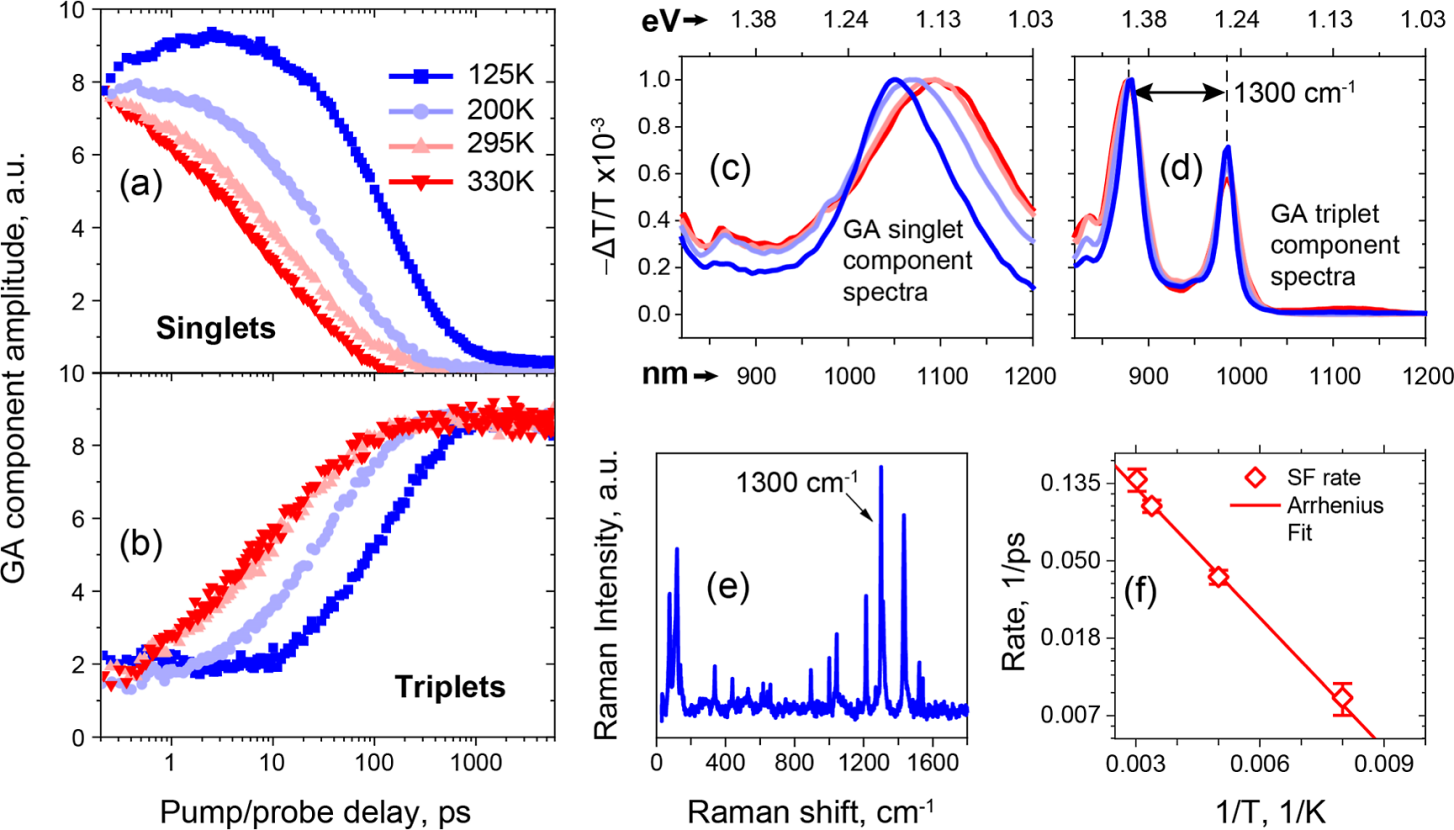
Kinetics and spectra of singlets (a,c) and triplets (b,d) from the global analysis (GA) of the temperature-dependent transient absorption spectroscopy of rubrene single crystals (430 nm excitation). Raman spectra of a rubrene single crystal measured with the excitation polarization directed along the 0*B* axis (e). SF rates in rubrene single crystals as a function of inverse temperature (f).

The magnitude of the barrier between relaxed **S** and **[S/TT]** (activation energy *E*_a_) can be estimated by applying the Arrhenius model to the fission dynamics. As shown in Figure 3f, the logarithm of the fission rate is inversely proportional to temperature over the entire measured range, indicating the thermally activated model is appropriate. Our fit yields an activation energy of 48 ± 3 meV, which is in reasonable agreement with values previously reported for more disordered rubrene systems.^14,44,46,47,76^

Figure 4a presents a state model of SF in ultrapure single-crystalline rubrene based on our TAS findings, which reconciles the existing literature data. Excitation with above-optical-gap photons leads to direct formation of mixed **[S/TT]** states. **[S/TT]** are partially bright states as they derive transition dipole moment from bright **S**.^25^ Having both **S** and **T** character, the **[S/TT]** states allow transitions into the **T**_***n***_ and **S**_**n**_ manifolds and thus possess both **S** and **T** spectral signatures in their transient absorption.^24^ The **T** → **T**_***n***_ transition is, however, red-shifted in **[S/TT]** compared to **T** due to the multiexciton stabilization energy.^11^ In pentacene derivatives, **[S/TT]** mixing was previously shown to be promoted by the overlap of **S** and **TT** vibronic manifolds and we believe this mechanism is also present in tetracene derivatives including rubrene.^9^

**Figure 4:**
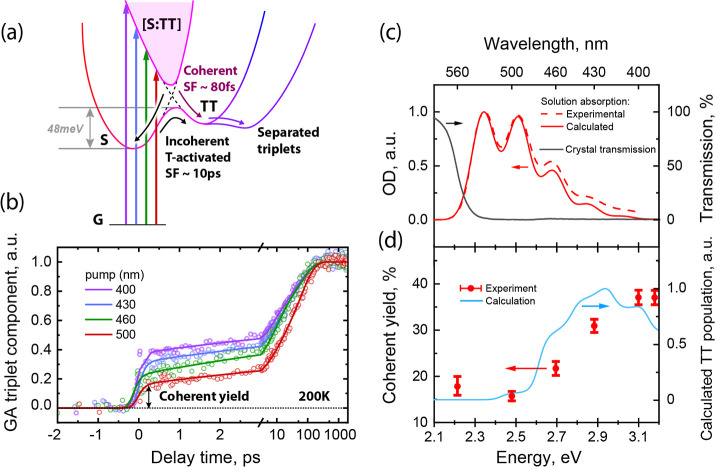
(a) SF model for rubrene single crystals. Tuning of the excitation wavelength allows ultrafast control of the triplet states generated immediately after the excitation (coherent SF). Thick blue lines indicate photoexcitation with different pump wavelengths; curved black and pink lines point to internal interband transitions. (b) Triplet global analysis (GA) component kinetics dependence on the pump wavelength measured at 200 K. (c) Absorption spectrum of rubrene molecules in deuterated chloroform solution as calculated with the 3-state model and comparison with experiment. Experimental transmission spectrum of the crystal is shown for reference. (d) Relative increase in **TT** state population as a function of excitation pulse energy compared with the calculated coherent triplet yield. Red dots with error bars are experimental points derived from triplet GA component kinetics (left axis).

Within the first ∼100 fs after photoexcitation, the initially formed **[S/TT]** mixed states relax to either predominantly triplet-pair **TT**, or to “pure” singlet states **S** (we estimate ∼1.5 times more relaxation into **S** compared to **TT**, based on the relative amounts of prompt SF). This exothermic process is the first, fast, and most likely coherent SF pathway in rubrene. Cooling to the pure **S** is followed by slow (>10 ps) thermal activation of singlets over the 48 ± 3 meV barrier back to **[S/TT]** and further to triplet states. Our data show that the rate of this second process is limited by thermal activation and we do not detect any further intermediates prior to **T**. Therefore, the conversion of **TT** to separated triplets **T** likely occurs on a faster time scale than 10 ps. In our optical experiments, we have not identified any distinct differences in the TA signal of exchange-coupled triplet pairs **TT** or free triplets **T**. However, recent literature reports on the exchange coupling of **TT** states (<5 meV) in a similar acene material TIPS-tetracene^77^ support that our assumption about the fast conversion of the **TT**-pairs intro free triplets states **T** is reasonable.

Crucially, the presented model suggests a simple way to control the branching between coherent and incoherent SF pathways. The initially excited states must be bright and therefore have substantial **S** character. However, **S** and **TT** states exist in a dense vibronic manifold and one can expect that the higher-lying states will have higher degree of mixing and higher contribution of **TT** character. This higher triplet character should increase the probability for the mixed state to branch off directly to pure **TT** via the coherent fission pathway. Indeed, we find that excitation with higher photon energy increases the relative contribution of the coherent pathway and decreases that of activated fission via **S** (Figure 4b,d). While the general dynamics of the two SF pathways stay the same, importantly, the contribution to the total triplet yield from the ultrafast coherent process changes from 18.0 ± 2.0% to 37.0 ± 1.5% over our pump energy scan. We also note the presence of coherent **TT** yield for the band-edge excitation, which we attribute to the small **TT** and **S** energy barrier being comparable to the spectral width of the excitation pulse.

Here the ∼40% contribution was estimated from the triplet kinetics offset measured with the 200 fs TAS setup at 200 K to enhance the separation of time scales between the coherent and incoherent fission pathways. This estimate agrees well with the one observed with the 14 fs TAS setup shown in Figure 2, which confirms the reliability of the stitching procedure of the data obtained from 3 different setups. This also implies that the coherent generation of the triplets, resultant of the mixed-state excitation, is not a consequence of using a spectrally wide and temporally short pulse creating coherent superposition of states in the system.

The analysis given above is further supported by the results of electronic-structure calculations and vibronic modeling. We first estimated the reorganization energy related to the **S** → **TT** transition. This energy can be roughly approximated as the sum of the relaxation energies associated with the **G** → **T** and **S** → **T** electronic transitions, where **G** denotes the ground state. The calculations performed at the density functional theory (DFT) level with the global hybrid B3LYP functional and 6–31G­(d,p) basis set yield a reorganization energy of about 0.3 eV. However, our previous studies^78^ have shown that B3LYP-based calculations usually underestimate the relaxation energies by up to 30%. In addition, we neglected the contribution to the reorganization energy due to the interactions with surrounding molecules; for nonpolar systems, this contribution is typically in the range of 0.1–0.2 eV.^79^ Taking all these factors into account, the reorganization energy related to the **S** → **TT** transition is estimated to be in the range of 0.4–0.6 eV. If we were to follow the classical Marcus theory, such values would lead to an activation barrier between the relaxed **S** and **TT** states on the order of 100–150 meV, i.e., two-to-three times larger than the experimental 48 meV value. However, the DFT calculations indicate that the majority of the reorganization energy is due to nonclassical (high-frequency) vibrational modes, which calls for the application of the expanded Marcus–Levich–Jortner (MLJ) model. Using this model, taking into account the DFT-derived vibrational couplings, and assuming a contribution of 0.1 eV to the classical reorganization energy due to the surroundings, we estimate (Section XI of Supporting Information) the activation energy to be 52 meV, in very good agreement with experiment. In order to reproduce the experimental rate constants, our calculations have to assume a relatively small value of 7 meV for the electronic coupling between the **S** and **TT** states, which was obtained from the fitting of the Marcus–Levich–Jortner equation (Section XI of Supporting Information).

To describe the hybridization of high-energy vibrational modes of the **S** and **TT** states, we consider a fully quantum-mechanical vibronic model^80^ based on three electronic states (**S**, **TT**, and **G**) and two effective vibrational modes. This model treats the electronic and electron–vibrational interactions exactly^80^ (Section XI of Supporting Information). Figure 4c,d presents the results of the vibronic simulations for the rubrene absorption spectrum and the contribution of the triplet **TT** state in the **[S/TT]** mixed state populated by photon absorption as a function of photon energy. Our model shows that, as a result of a subtle interplay between electronic and electron–vibration interactions, the degree of direct (coherent) generation of the **TT** state in rubrene (where the reorganization energy is large) increases when photoexciting higher-energy **[S/TT]** vibronic sublevels.

Although our vibronic model captures well the trend observed experimentally (Figure 4d), we expect that a better agreement with the observed data could be achieved if additional electronic states were included into the model. We note, for instance, that the ionization energy and electron affinity of rubrene in the solid state are 5.4 and 2.7 eV, respectively.^81,82^ These values suggest that, when considering the electrostatic interactions between hole and electron, the lowest **CT** state in rubrene will appear at an energy lower than [5.4–2.7 = 2.7 eV]. Thus, the vibrational levels of the **CT** state could mix with the excited vibrational manifold of the **S** and **TT** states and contribute to **TT** generation or introduce some **CT** character to **TT**. We believe that the use of new methods, such as angle-resolved photoelectron spectroscopy, is a promising direction for future research, which may clarify this mechanism and reveal potential new properties of **[S/TT]** states.^35^ In our current model, the role of the **CT** states is accounted for indirectly to mediate the electronic coupling between **S** and **TT**, which otherwise would be zero due to symmetry constraints. Clearly, the extension of the present vibronic model to include explicitly the **CT** states would provide a deeper understanding of the formation and branching of the **[S/TT]** states. We expect that, due to the relatively higher energy of the **CT** states, their effect should be especially pronounced at high photoexcitation energies with wavelength <460 nm.

Several previous studies have suggested that the singlet fission process in some material systems involves a combination of “coherent” and “incoherent” pathways, though the (in)­coherent nature of the process is sometimes ambiguously defined. Typically, both pathways are based on a vibronic coupling mechanism.^15^ Uniquely in our study, we present evidence that, following direct excitation, a mixed **[S/TT]** state is immediately formed. The “coherent” channel in this context can be accurately defined as an ultrafast demixing of the initial **[S/TT]** state. The fraction of the population projected into the pure **S** state then evolves through an incoherent, thermally activated singlet fission pathway to the same terminal **TT** state.

Our work potentially resolves the controversy between the two SF models for crystalline rubrene that have been debated in the literature. Coherent SF is occurring immediately after photoexcitation, as highlighted for example by Miyata et al., and the incoherent (hopping-like) SF is taking place over a potential energy barrier via thermal activation.^14^ According to the coherent SF model, an electronic wave packet formed by photoexcitation may evolve into either cold singlet or triplet-pair states via a conical intersection of the **S** and **TT** potential energy surfaces enabled by a symmetry-breaking mode; this was corroborated by the experimental observation of temperature-independent step-like PIA at 510 and 800 nm in the TAS kinetics, attributed to a fast (sub-100 fs) formation of triplets. Our results suggest that the step-like signals may be coming from the mixed states, which possess both **S** and **T** TAS signatures. In recent studies,^83,84^ the manifestation of the step-like signals was associated with the defects in crystals, which may locally break crystal symmetry and facilitate SF. However, in this work we found that coherent SF is observed in ultrapure crystals, which were carefully screened with a range of techniques including PL, FET charge transport, and Hall-effect mobility measurements (Section I of Supporting Information). The use of high-quality samples, high temporal resolution, and wide probe range allowed us to differentiate **TT** and **[S/TT]** states and identify individual states in the dynamics. We note that another explanation of the rapidly appearing triplet signal has been proposed by Turner et al. in the framework of incoherent SF;^40^ according to that model, the rapid observation of triplet pairs with 2D electronic spectroscopy is related to weakly coupled but nearly resonant electronic energy levels of the singlet and triplet-pair states. However, this proposition, despite its attractive simplicity, is unable to explain the temperature-dependent fission rates. The scenario we put forward resolves the contradictions among the various theories by showing that both ultrafast exothermic and slow endothermic SF pathways stem from the **[S/TT]** state.

## Conclusion

To conclude, with muti-time scale TA spectroscopy we have tracked the SF dynamics in high-purity rubrene single crystals, from photoexcitation (∼14 fs) to triplet exciton decay (∼μs). Our experiments revealed that SF in rubrene begins with the formation of a very short-lived state with a hybrid singlet/triplet character, which rapidly (∼80 fs) coherently decays into either a singlet state or separated triplets **T**. This hybrid state can be described as a vibronically enhanced mixture of the **S** and **TT** multiexciton states. Beyond reconciling the disparate models of singlet fission in crystalline rubrene, our observation of direct excitation of a mixed **[S/TT]** state points to new ways in which SF can be optimized in endothermic systems. Here, we have demonstrated the SF pathway control via the choice of excitation photon energy. Further work may target different molecular design aspects, such as intermolecular couplings and the density of vibronic manifold, to achieve a better control of the fission mechanism and the realization of SF-enhanced photovoltaic materials.

## Supplementary Material



## Notes

Experimental methods, matching the crystal-axis orientations with the habitus of rubrene single crystals, TA spectroscopy of amorphous Rubrene films, TA spectroscopy of coherent beating signals and their Fourier analysis, Widths and positions of singlet and triplet spectral components as a function of temperature, power dependence of TA kinetics, matching TA data measured with different setups, the full set of triplet SF kinetics as a function of pump wavelength, anisotropy effects on SF kinetics and spectra, dependence of coherent triplet yield on temperature, computational methodology (PDF)
