# Supplementary material for: Interplay between Mixed and Pure Exciton States Controls Singlet Fission in Rubrene Single Crystals
Source: J Am Chem Soc. 2025 Jun 24;147(27):23536–44. doi: 10.1021/jacs.5c02993 (PMC12257525; doi:10.1021/jacs.5c02993)
Supplement: Supplementary file 1 [file ja5c02993_si_001.pdf]

Interplay Between Mixed and Pure Exciton States Controls Singlet Fission in Rubrene Single Crystals

Supporting Information

Dmitry R. Maslennikov[1], Marios Maimaris[1], Haoqing Ning[1], Xijia Zheng[1], Navendu Mondal[1], Vladimir V. Bruevich[2], Saied Md Pratik[4], Yifan Dong,[1] John W.G. Tisch,[5] Andrew J. Musser[3], Vitaly Podzorov[2], Jean-Luc Bredas[4], Veaceslav Coropceanu[4], and Artem A. Bakulin[1]*

[1] *Department of Chemistry and Centre for Processible Electronics, Imperial College London, UK*
[2] *Department of Physics and Astronomy, Rutgers University, New Jersey, USA*
[3] *Cornell University, Department of Chemistry and Chemical Biology, USA*
[4] *Department of Chemistry and Biochemistry, The University of Arizona, Tucson, USA*
[5] *Department of Physics, Imperial College London, London, UK*
* a.bakulin@imperial.ac.uk

Table of Contents

Table of Contents 2

Section I. Methods 3

1.1 Single crystal growth 3

1.2 Evaluating quality of the rubrene single crystals 3

Steady state photoluminescence spectroscopy 3

Charge transport measurements 4

1.3 Transient absorption (TA) 7

Sub-14fs setup and pulses characterization 7

200-fs setup 10

Ns setup 11

Section II. Matching the crystal-axis orientations with the habitus of rubrene single crystals 12

Section III. TA spectroscopy of amorphous Rubrene films 13

Section IV. TA spectroscopy of coherent beating signals and their Fourier analysis 14

Section V. Widths and positions of singlet and triplet spectral components as a function of temperature 18

Section VI. Power dependence of TA kinetics 19

Section VII. Matching TA data measured with different setups 20

Section VIII. The full set of triplet SF kinetics as a function of pump wavelength 21

Section IX. Anisotropy effects on SF kinetics and spectra 22

Section X. Dependence of coherent triplet yield on temperature 23

Section XI. Computational methodology 24

Vibronic model 33

Supplementary References: 36

Section I. Methods

1.1 Single crystal growth

Rubrene single crystals were grown by multiple recrystallizations of a rubrene powder purchased from GFS Chemicals. To investigate the intrinsic photo-physical properties of rubrene single crystals, we grew the crystals by the best way available to us: a physical vapor transport (PVT) growth in a stream of ultra-high purity He gas[1,2], at about 1 atmosphere and a flow rate of ~ 100 sccm. The temperature in the sublimation zone was about 320 °C. Depending on the amount of the material loaded in the furnace (typically 250 – 500 mg of rubrene powder), the duration of each growth was 24-48 h. For achieving the best purity, 3-5 stages of recrystallization were carried out. The crystals grown in this manner and under the same conditions have been already thoroughly evaluated for purity, as well as investigated to obtain their intrinsic transport parameters (i.e., the intrinsic hole mobility).[3] As an indirect yet very efficient method of evaluating the quality of the crystals, we have fabricated field-effect transistors (FETs) and performed FET and Hall-effect measurements showing intrinsic charge transport (with fully developed Hall effect) and high field-effect hole mobility (see Section 1.2 of SI).

1.2 Evaluating quality of the rubrene single crystals

Steady state photoluminescence spectroscopy

To characterize the quality of the crystals measured in this work we performed PL and carrier mobility measurements. The photoluminescence of organic solids, even in their highly purified single crystalline form, are very sensitive to trace impurities and structural defects.[4–6] Even tiny concentrations of impurities[7] have been shown to affect the PL spectra of organic SCs. Fig. S1b shows the representative PL spectrum of the single crystal used in temperature-dependent TAS measurements. We found this spectrum to be very close to the spectra of pristine rubrene single crystals reported in the literature with the dominant band at ≈610 nm. No redshift inherent to defective samples was observed[5].

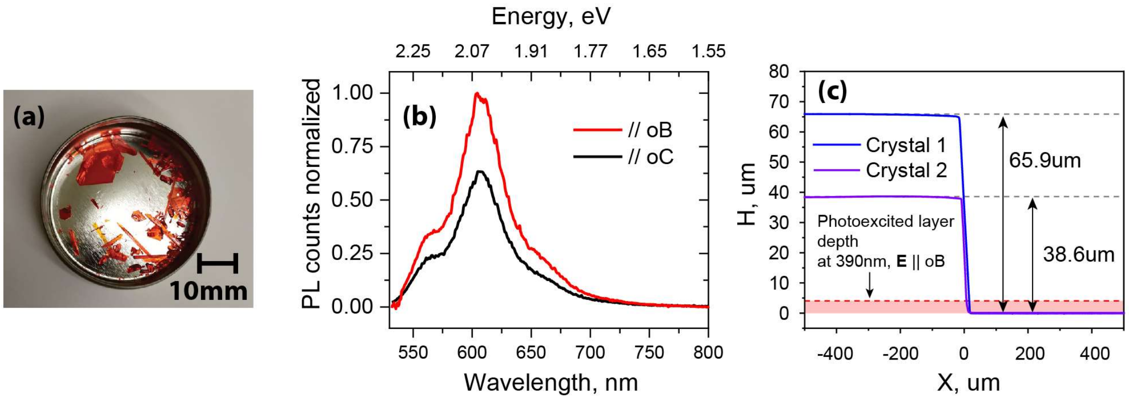

Fig. S1. (a) Rubrene single crystals after growth before transferring to substrates. In some cases, the crystal size exceeds 1 cm. (b) PL spectrum of a rubrene SC sample that was later used in transient absorption spectroscopy measurements. The crystal was photoexcited with a 405 nm diode laser with a beam size of ~ 0.5 mm². (c) Height profiles of the Rubrene single crystals measured with a profilometer in comparison with a maximal penetration depth of pump light at 390nm (maximal depth for this study). Photoexcitation depth was calculated as (1/α), where α is the extinction coefficient (see Figure 1 in the main text). Crystal 1 was used for transient absorption measurements in this study; crystal 2 is another representative Rubrene single crystal from the same growth batch.

Charge transport measurements

The charge carrier mobility, μ, of crystalline organic semiconductors is usually very sensitive to the so-called static disorder (e.g., defects and chemical impurities incorporated in the crystal structure). Rubrene single crystals grown via the physical-vapor transport (PVT) method are well suited for the fabrication of high-performance field-effect transistors (FETs), because an efficient purification of the material and a great morphology of the crystals can be achieved after multiple re-crystallization cycles.[1] The FET mobility in such devices depends on several important intrinsic factors (e.g., the intrinsic molecular packing and transfer integrals, the off-diagonal thermal disorder and carrier momentum relaxation time, as well as the dielectric permittivity of the gate dielectric). However, the mobility is also influenced by such extrinsic factors as the surface/interface roughness and the density of traps and scattering centres at (or near) the interface. In order to assess the structural order and purity of the crystals, one can take

advantage of the so-called charge carrier coherence factor, α (see, e.g., Ref. [8]), together with the absolute values of the FET and Hall carrier mobilities, μ_{FET} and μ_{Hall}. These parameters help evaluate the overall quality of the FETs and judge the level of static and dynamic disorder in the semiconducting material. The carrier coherence factor is defined as: $\alpha \equiv \mu_{\mathrm{Hall}}/\mu_{\mathrm{FET}}$, where the mobilities are determined via the Hall-effect and the longitudinal FET measurements. In the case of relatively high carrier mobilities ($\mu > 1\, cm^{-1}V^{-1}s^{-1}$), $\alpha \approx 1$ would signify a band-like charge transport mostly dominated by band (delocalized) carriers, with an insignificant contribution from hopping carriers.[8] Thus, evaluating μ_{FET}, μ_{Hall}, and α simultaneously is a good way of getting a sense of the material's purity.

As described in the main text, rubrene single crystals used in the optical measurements reported in this paper were grown by carrying out multiple PVT recrystallization cycles (typically 3-5). Such a methodology results in a gradual purification of the organic material, eventually leading to the best performing FETs.

The electrical characterization of such crystals has been carried out using FET and Hall-effect measurements, as presented in Fig. S1. Panel (a) shows the transfer characteristics (i.e., the source-drain current vs the gate voltage, $I_{SD}(V_G)$) of a single-crystal rubrene FET measured in the linear regime (that is, at $|V_{SD} < |V_G - V_T|$, where V_{SD} is the source-drain voltage, V_G is the gate voltage, and V_T is the threshold voltage). Panel (b) shows the resultant FET mobility, μ_{FET}, and the Hall mobility, μ_{Hall}, both obtained in the transistor's linear regime using the four-probe measurement technique.[9] The charge carrier coherence factor, α, calculated from these data is also plotted. The FET mobility, μ_{FET}, has been obtained using the Shockley's FET model for the linear regime:

$$\sigma \equiv \frac{I_{SD}}{V_{4p}} \cdot \frac{D}{W} = \mu_{\mathrm{FET}} \cdot C_i \cdot (V_G - V_T - V_{SD}/2), \qquad \text{(S1)}$$

where σ_{4p} is the contact-corrected (four-probe) channel conductivity per square (in Ω^{-1}), C_i is the gate-channel capacitance per unit area (for this device, C_{i} = 1.41 nF·cm^{-2}), V_{4p} is the four-probe voltage measured between the two voltage probes located in the middle of the channel, *D* is the longitudinal separation between these probes, and W is the channel width. The threshold voltage has been determined via the Hall measurements, and it was close to zero ($V_{\mathrm{T}} \approx 0$). We note that μ_{FET} was obtained by directly applying equation (S1) to the data shown in Fig. S1(a),

without taking the $\frac{\partial I_{SD}}{\partial V_G}$ derivative. The Hall mobility, μ_{Hall}, was measured using the high-sensitivity *ac*-Hall technique, with a magnetic field oscillating at a frequency f = 0.6 Hz. The details of the *ac*-Hall methodology can be found elsewhere.[3,10]

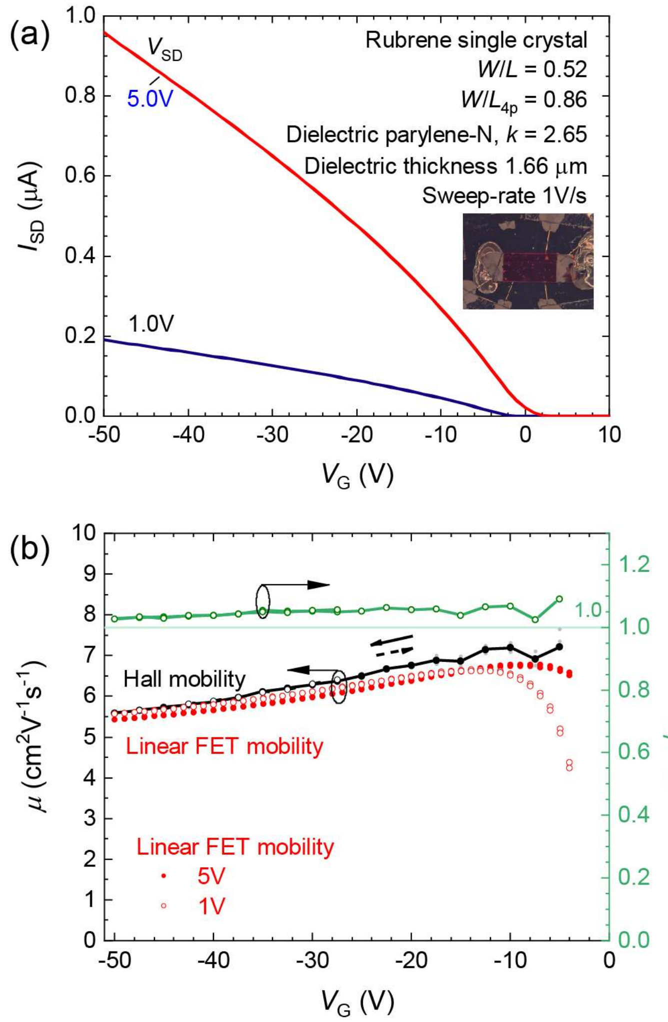

Fig. S2. Electrical characterization of FETs based on high-purity rubrene single crystals analogous to those used in the optical measurements reported in the main text. (a) The FET's transfer characteristics measured in the linear regime (the device parameters and a photo are given as insets). (b) The FET and Hall carrier mobilities, μ_{FET} and μ_{Hall} (left axis), and the calculated charge carrier coherence factor, $\alpha \equiv \mu_{Hall}/\mu_{FET}$ (right axis), extracted for this device.

The carrier coherence factor $\alpha \approx 1$ indicates that the influence of hopping carriers on the overall change transport is very small, thus implying a negligible concentration shallow traps in the transistor's channel (the so-called *intrinsic* charge transport regime). A very small threshold voltage $V_T \approx 0$ is indicative of a negligible concentration of deep traps. Such behaviour of the FET devices attests to a very high purity and structural order of the single crystals used in their construction.

1.3 Transient absorption (TA)

To achieve both high temporal resolution and broad time range, TA of rubrene single crystals was measured using three different setups with different time resolution and pump/probe delay range and then stitched together according to the same features in the overlapping time delay regions. The sub-14fs resolution setup used for measurements (<10ps) is based on the chirped mirrors compressor system described in ref. [[11]]. A commercially available TA spectrometer Helios (Spectra Physics, Newport Corp.) was used to measure the TA in the intermediate time delay region (>0.2ps, <6ns). Finally, a home-built TA spectrometer in conjunction with a q-switch nanosecond laser (Picolo, InnoLas) was used for TA measurements >1ns. For Figure 2 in the main text, we used 400nm (50nJ), 430nm (160nJ) and 532nm (75nJ) pump excitation correspondingly focused on the samples with a beam size of around 0.5 mm^2. As a reference for the wavelength scale, we used the intermediate time delay dataset (>0.2ps, <6ns) collected with the commercial spectrometer. Relative intensity of the triplet peaks in >1ns region was adjusted to match intermediate time delay dataset (Figure 2a). To balance the signal coming from the PIA of singlet and triplet excitons, probe polarization was oriented approximately 60° relatively to the 0B crystal axis for the fastest (<10ps) and intermediate (>0.2ps, <6ns) setups measurements. A liquid nitrogen cryostat (OptistatDN-V, Oxford Instruments) was used for the temperature control.

Sub-14fs setup and pulses characterization

Fig. S3 shows the experimental apparatus for sub-14-fs TA. A commercial hollow-fibre pulse compression (HFPC) system (Imperial Consultrants) was used to generate sub-14-fs pulses from a 800 nm, 50 fs Ti:sapphire laser (Coherent Astrella, 2.5 W, 4 kHz pulse repetition rate). The 50 fs pulses, at an average power of 1.25 W (~300 μJ), were coupled into a 250 μm diameter differentially-pumped hollow-core fibre (evacuated with a vacuum pump at the fibre entrance

and filled with argon at 0.9 bar at the fibre exit) to spectrally broaden the pulse, thus permitting pulse temporal compression. The HFPC system uses beam-pointing stabilisation to lock the position and angle of the input beam to the entrance of the fibre to ensure the long-term stability required for lengthy data scans. The output pulses from the fibre have photon energies in the range 1.4-1.9 eV (650-900 nm). Temporal compression to ~10-fs duration was achieved via dispersion compensation from 10 bounces off chirped mirrors (Layertec, 40 fs^2 per bounce). The compressed pulses were split approximately 50/50 into two arms using a d-cut mirror. The first arm is the probe/push arm where the sub-14-fs pulse serves as the probe/push pulse and passes through a micrometre-precision delay stage (LNR50 Series Encoded, Linear, Long-travel Translation Stage, Thorlabs) to control the time delay between the pump and probe/push pulses. The second arm is the pump arm where a 50 μm BBO crystal is used to generate a pump pulse centred around 3.1 eV (400 nm) using Type-I second harmonic generation. Another set of chirped mirrors (Ultrafast Innovations, 50 fs^2 per bounce) was used to compensate the 400 nm beam for dispersion in the BBO crystal and the air path, resulting in sub-14-fs pump pulses. A waveplate was inserted before the d-cut mirror to ensure that the polarisation of the second harmonic was orientated correctly for the pump chirped mirrors. The dispersion in each of the arms is independently optimised by fine tuning the amount of glass in the optical path via two sets of AR coated glass wedges mounted on translation stages. The two beams (pump and probe/push) were then recombined using a second d-cut mirror and focused into the sample-device with a f = 30 cm focusing mirror using a small angle of incidence (<5°) to minimise astigmatism. Diode array detected the transmitted probe pulse for TA experiments. Reflective optical components were used to avoid additional dispersion compensation and to preserve the temporal resolution.

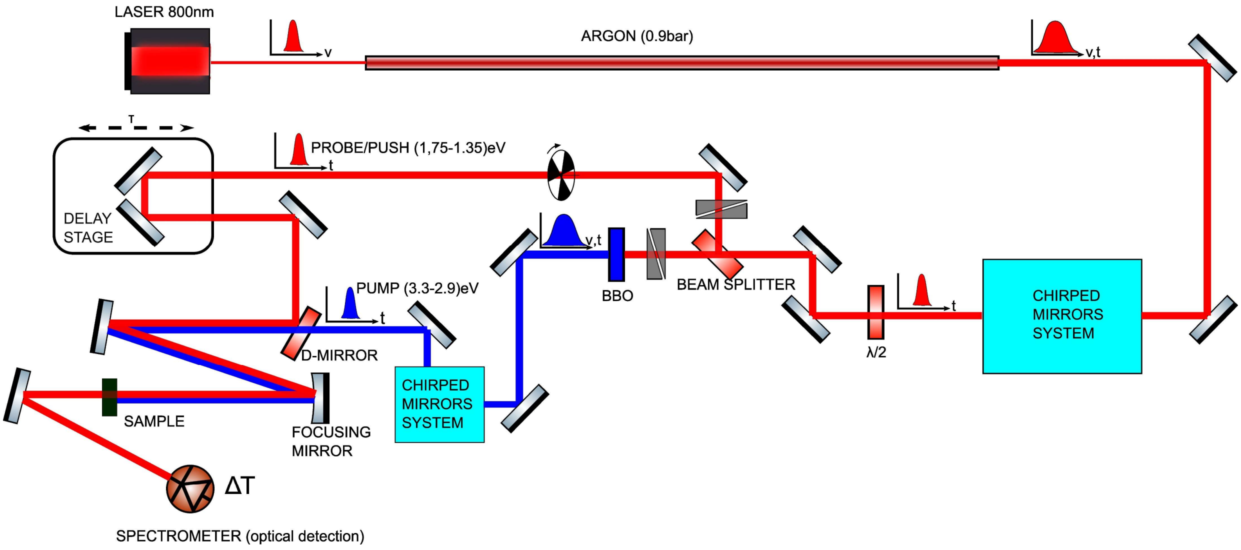

Fig. S3. Experimental apparatus of sub-14fs TA experiments.

Fig. S4 shows the spectra of pump and probe pulses used in this study and generated with the above experimental apparatus. The pump pulse is ~2000cm^{-1} broad and has solid Gaussian-like shape. The probe pulse is broader but suffers from a modulation which probably makes it the limiting factor for time resolution. To characterise the probe pulse we measured autocorrelation of two probe pulses in the tunnelling junction[12] (Fig. S5a) and received the autocorrelation FWHM of ~18fs corresponding to roughly 12 fs pulse duration, including possible chirp.

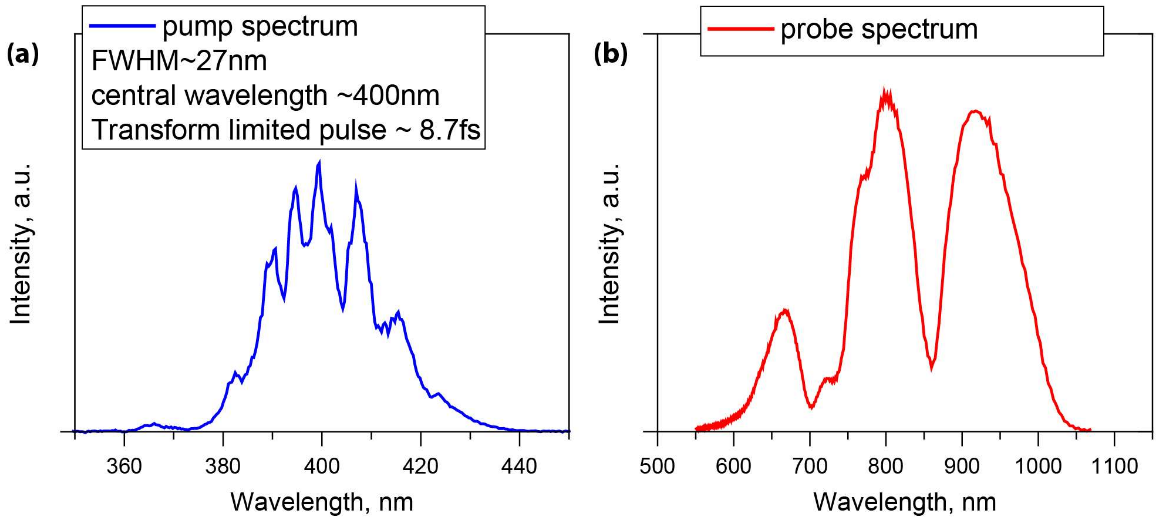

Fig. S4. (a) Normalised pump (blue) and (b) probe/push (red) pulses spectra.

To estimate time resolution in the most direct way, we measured the TA response in a polyfluorene (PFO) film, which was previously shown to have a large 'instant' component[11] (Fig. S5b). To reproduce the signal build-up, the TA transient can be fitted by a convolution of an instant response with a 14fs gaussian function. This gives 14 fs as a good estimate for the time resolution and is consistent with having slightly chirped 12-fs probe and sub-10fs pump.

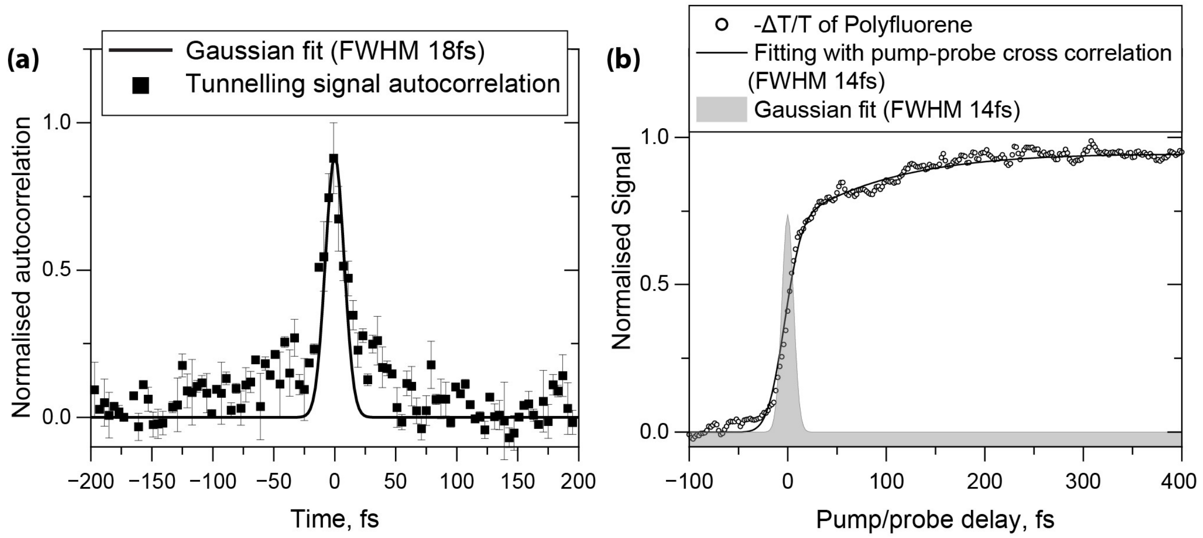

Fig. S5. (a) Autocorrelation of the probe pulse measured using a tunnelling signal in a nanojunction. (b) TA response measured in a polyfluorene sample and analysis of the 'instant' growing component.

200-fs setup

A commercially available broadband pump-probe femtosecond (fs) transient absorption spectrometer Helios (Spectra Physics, Newport Corp.) was used to measure the transient absorption in the intermediate time delay region (>0.2ps, <6ns). Ultrafast laser pulses (800 nm, 100 fs duration) were generated by a 1 kHz Ti:sapphire regenerative amplifier (Solstice, Spectra Physics, Newport Corp.). One portion of the 800 nm pulse was directed to an optical parametric amplifier (TOPAS Prime, Spectra-Physics) and a frequency mixer (Niruvis, Light Conversion) to tune the visible pump pulses to 390 – 560 nm. The pump pulses were modulated at a frequency of 500 Hz by a mechanical chopper. The rest of the 800 nm pulse was routed onto a mechanical delay stage with a 6 ns time window and directed through a non-linear crystal (YAG for the NIR region) to generate a white light probe ranging from 830 – 1600nm range. The probe pulse was

split into two by a neutral density filter. One portion of the probe pulse served as the reference and was directly sent to the fiber-optic coupled multichannel spectrometers with InGaAs CMOS sensor. The rest of the probe pulse together with the pump pulse were focused onto the same spot on the samples with a beam size of around 0.5 mm^2 before sending it to the spectrometer. To compensate the fluctuations, the measured spectrum was normalized to the reference spectrum and averaged for several scans to achieve a good signal-to-noise ratio. The pump pulse fluence was <50μJ/cm^2 to avoid non-linear processes. Coherent triplet yields (Figure 4d, main text) were extracted from the GA triplet kinetics (Figure 4b, main text) by fitting the data with a bi-exponential model convoluted with a Gaussian (σ = 0.1ps) to account for the instrument response function.

Nanosecond setup

The setup for ns measurement was analogous to the 200-fs setup, however the second harmonic of Q-switch ~0.8 nanosecond Nd:YAG laser (Picolo, InnoLas) was used as a pump. The pump laser was synchronized with Ti:Sapphire laser generating continuum probe using a delay generator (SRS DG645) which resulted in a ~5ns jitter, which determined the temporal resolution of the setup.

Section II. Matching the crystal-axis orientations with the habitus of rubrene single crystals

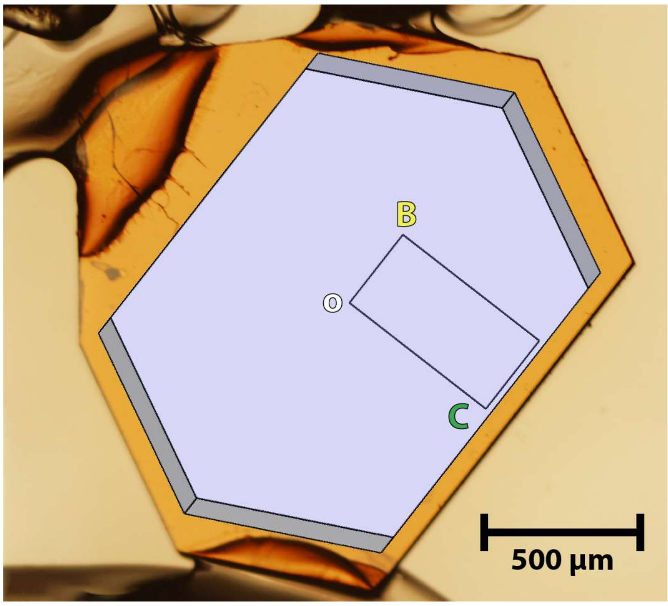

Fig. S6. Assignment of the single crystal habitus to unit cell orientation. Background – a rubrene single crystal microscopy image. Foreground – habitus model and corresponding orientations of the unit cell axes calculated with the Morphology module of the Material Studio package[13] *(Compass force field).*[14]

Section III. TA spectroscopy of amorphous Rubrene films

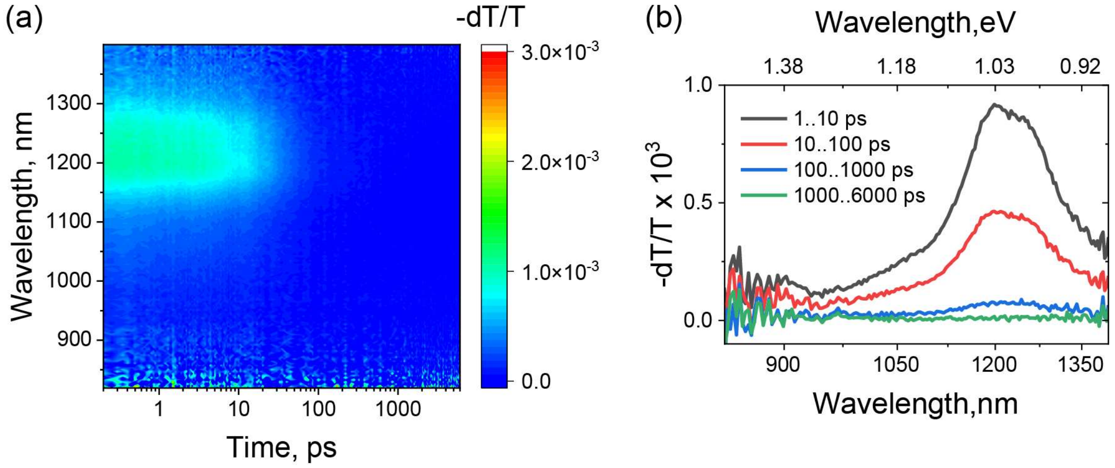

Fig. S7. Transient absorption of an amorphous rubrene film prepared from a toluene solution (10mg/ml). (a) 2D map measured at room temperature. (b) Spectra for several time ranges.

Section IV. TA spectroscopy of coherent beating signals and their Fourier analysis

During the TA measurements with high and low time resolution we observed coherent oscillations that we assigned to a superposition of vibronic states created during the fast fission process.

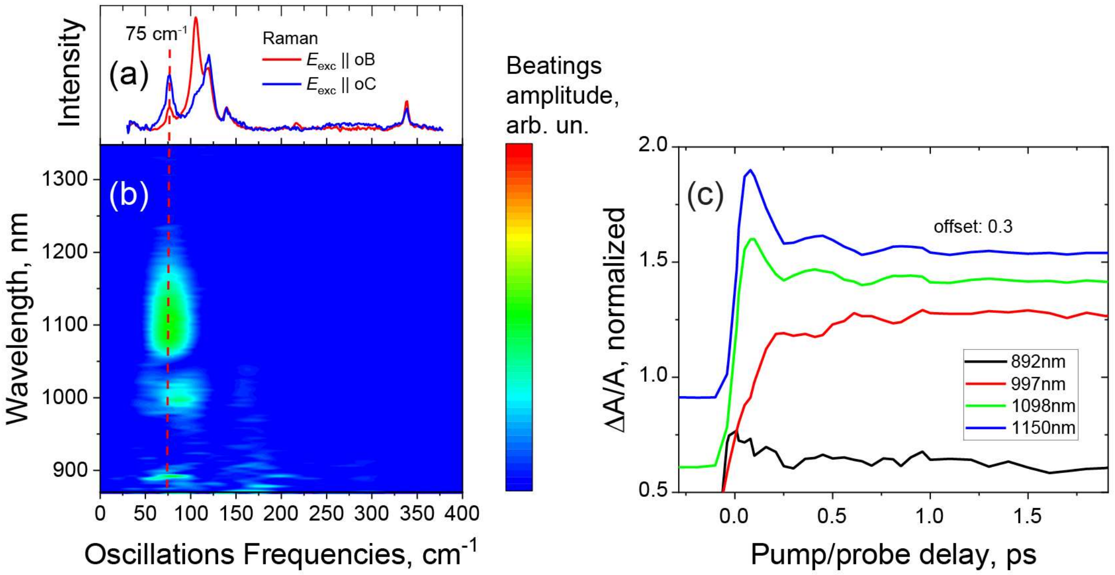

Fig. S8. Coherent oscillations in TA measured with 200-fs time resolution at room temperature. (a) Non-resonant Raman data used for the interpretation of the results. (b) Map of coherent beatings as a function of probe energy. (c) Representative time-domain traces at different probe wavelengths.

Fig. S8c presents the time-domain data recorded with ~200 fs resolution in ps-TAS setup. The oscillations were observed (Fig. S8b) dominantly in the wavelength region of singlet excited-state absorption (1060-1050 nm) and triplet excited-state absorption (980-1010 nm). This probe wavelength dependence confirms their identity as excited-state rather than ground-state coherences. The dominant peak is observed at 75 cm^{-1} and corresponds well to a mode clearly detectable in the non-resonant Raman spectrum (Fig. S8a).

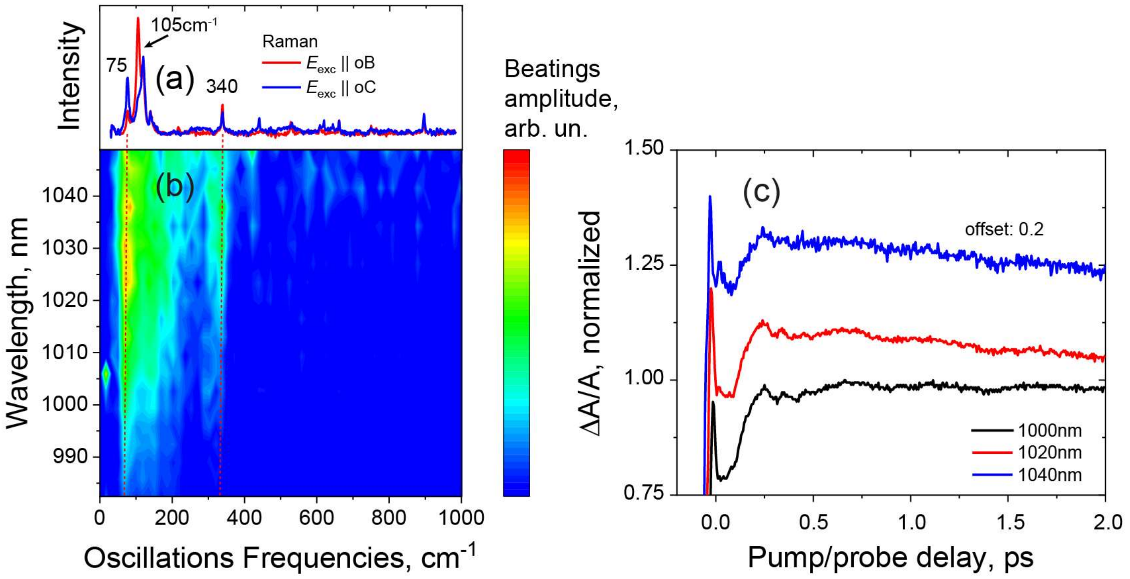

Fig. S9. Coherent oscillations in TA measured with 14-fs time resolution at room temperature. (a) Non-resonant Raman data used for the interpretation of the results. (b) Map of coherent beatings as a function of the probe energy. (c) Representative time-domain traces at different probe wavelengths.

Similarly, 14fs-TAS data (Fig. S9) reveal clear oscillations in the singlet excited-state absorption region as well as in the triplet excited-state absorption region (980-1000 nm). The spectrum of observed beating frequencies (Fig. S10) is clearly broader, particularly for the 'singlet' region. We observed additional strong modes at 120 cm^{-1}, 340 cm^{-1}, 420 cm^{-1} and some much weaker higher-frequency modes up to 900 cm^{-1}. All the beating frequencies are clearly identifiable in the rubrene Raman spectrum and are likely to be associated with excited-state vibrational coherences.

The fact that high-frequency modes are strongly suppressed, even when very short pulses are used, indicates that the actual timescale of generating superposition of states is slower than 14 fs, given our system capability of generating and detecting modes up to 2400 cm^{-1}. The 'cut off' frequency of 420 cm^{-1} indicates that singlet is impulsively populated on a time scale of 80 fs and triplet (**TT**) within a similar time or slower. This timescale agrees well with the decay time of the mixed **[S:TT]** state extracted from the global analysis of population dynamics.

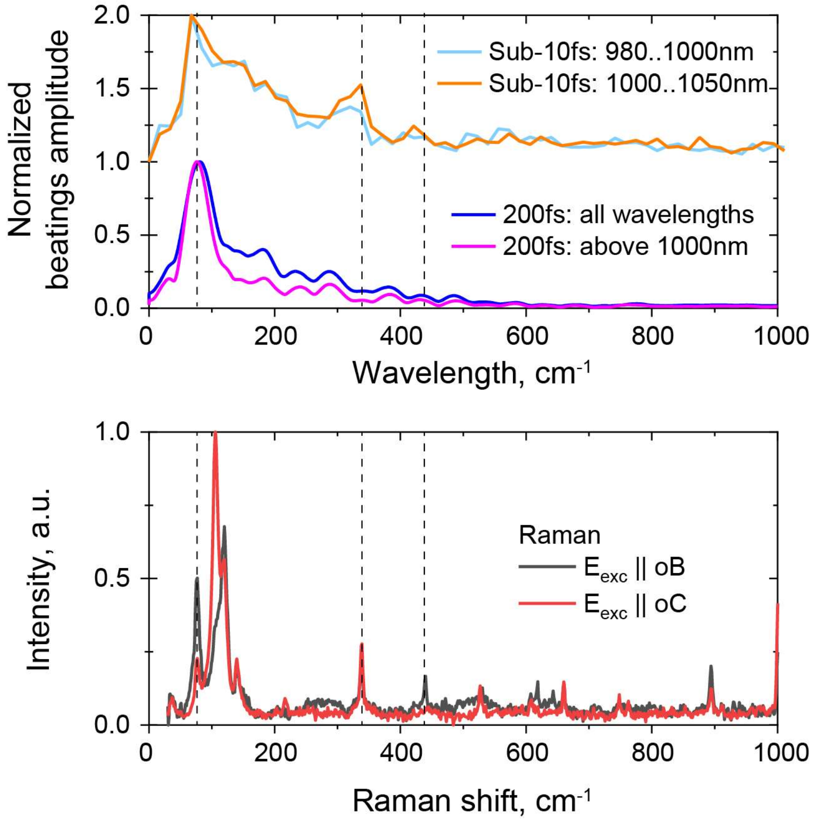

Fig. S10. Analysis of coherent oscillations in TA measured with 14-fs and 200-fs time resolution at room temperature. (top) Coherent beating spectra at different probe wavelengths. (bottom) Non-resonant Raman data used for the interpretation of the results.

We note on the origin of vibrational coherences observed in the transient absorption data. The observed modes appear exclusively in the PIA region and are limited to low frequencies (<400 cm^{-1}, Fig. S10). Such selectivity is not expected for coherences generated purely by impulsive excitation of ground-state modes, which should result in a broader frequency distribution. These observations support the assignment of the coherences to excited-state vibrational dynamics. While the comparison with steady-state Raman spectra offers useful insights, it is complicated in the low-frequency region due to broad linewidths, small shifts, and polarization-dependent selection rules. Therefore, only qualitative comparisons are made.

The absence of vibrational wavepackets above ~400 cm^{-1} in our transient data is not necessarily due to the lack of potential energy surface displacement along these coordinates.

Instead, it likely reflects the finite formation time (~80 fs) of the [S:TT] state. High-frequency coherent oscillations require both significant displacement and impulsive excitation, which in turn demands sub-80 fs formation dynamics to support the necessary bandwidth. The delayed population of the [S:TT] state thus limits the impulsive character of the excitation and suppresses coherence generation in the higher-frequency region.

Section V. Widths and positions of singlet and triplet spectral components as a function of temperature

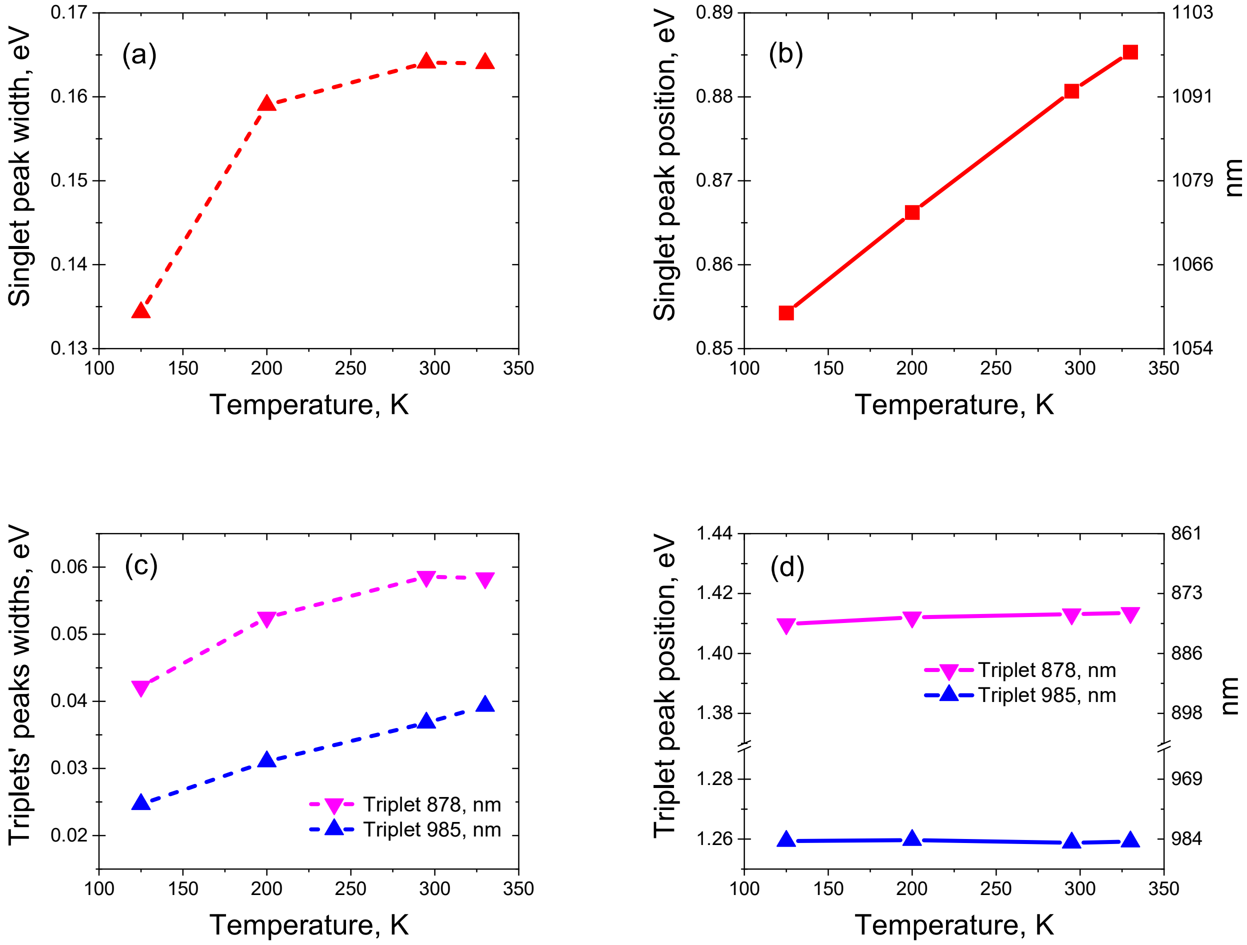

Fig. S11. Spectral signatures of singlet and triplet components as a function of temperature. Peaks widths (a, c) and peaks positions (b, d).

Section VI. Power dependence of TA kinetics

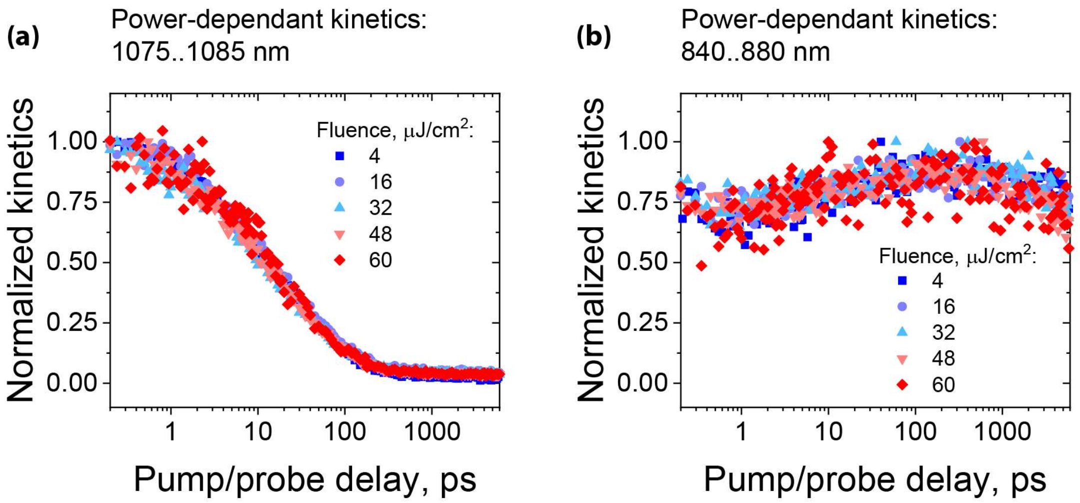

Fig. S12. Transient absorption fluence-dependant kinetics of a rubrene single crystal (normalized) (a) averaged from 1075 to 1085nm (wavelength region dominated by the singlet signal), and (b) averaged from 840 to 880nm.

Section VII. Matching TA data measured with different setups

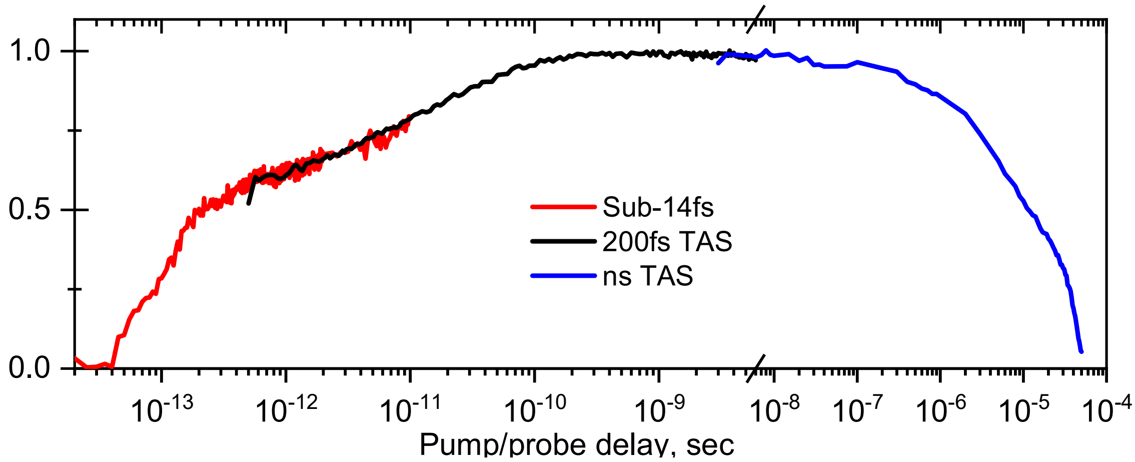

Fig. S13. Global analysis (GA) extracted triplet dynamics measured by three setups. The kinetics perfectly match in the overlapping time windows.

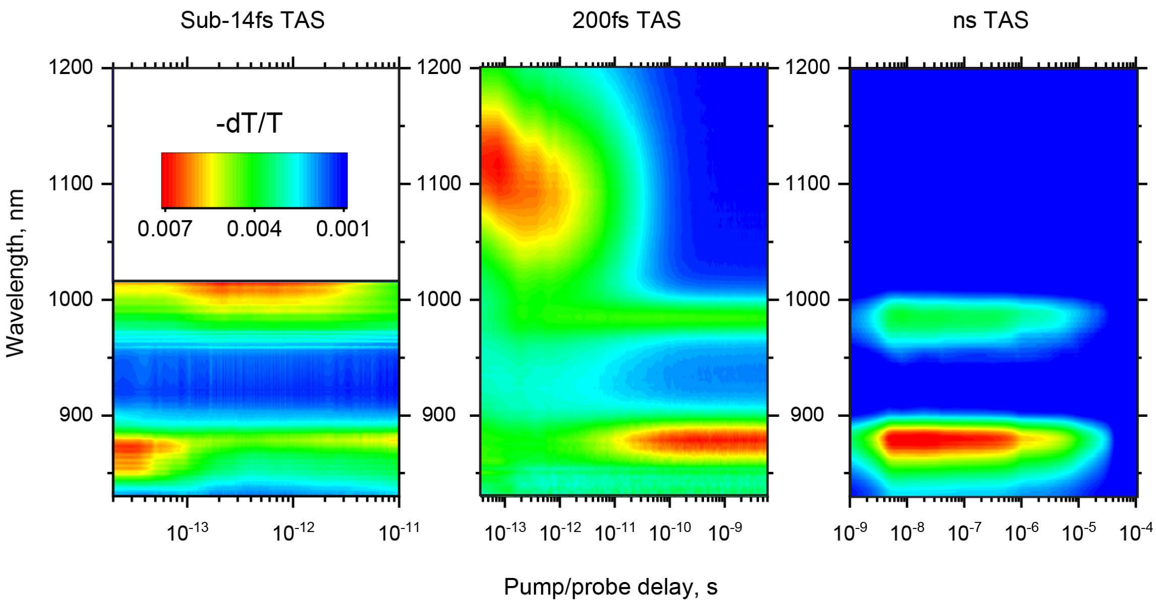

Fig. S14. Complete TA datasets measured by three experimental setups to produce figure 2 in the main text of the manuscript.

Section VIII. The full set of triplet SF kinetics as a function of pump wavelength

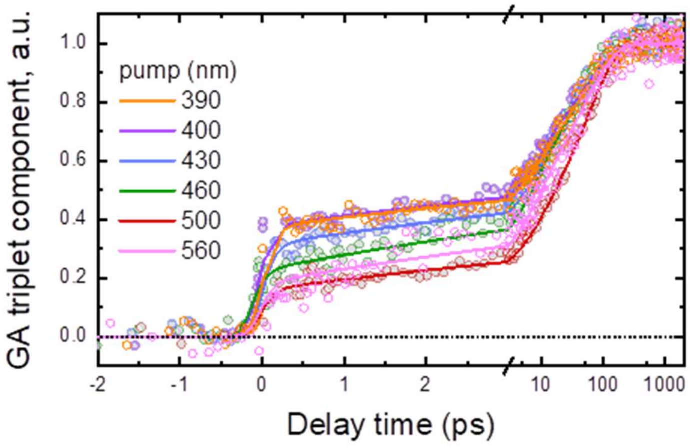

Fig. S15. The full set of triplet SF kinetics as a function of pump wavelength. This data were used to evaluate coherent fission contributions presented in Figure 4d in the main text.

Section IX. Anisotropy effects on SF kinetics and spectra

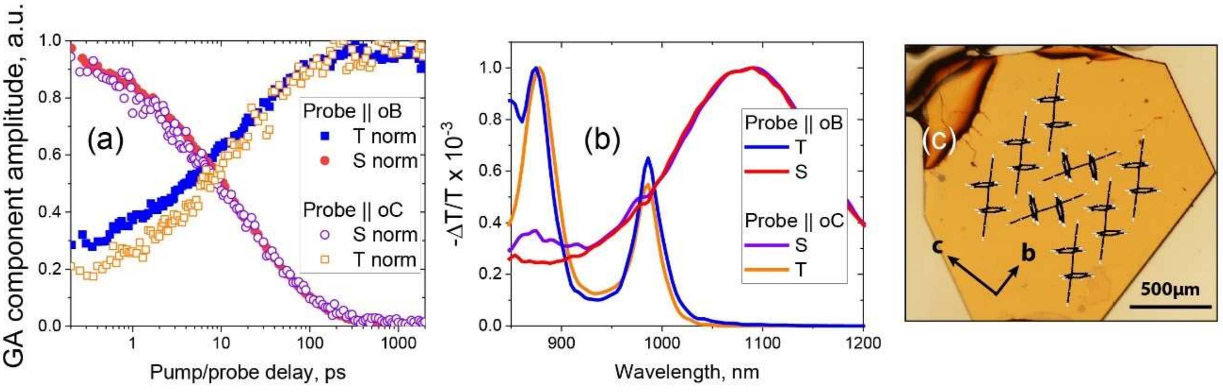

Fig. S16. The results of 200-fs TAS experiments with pump polarized along oB and oC axes of the crystal. The probe polarization was parallel to the pump. (a) Global analysis (GA) extracted population kinetics for triplets and singlets. (b) GA extracted spectra of triplets and singlets. (c) The crystal axes marked on an actual sample.

Section X. Dependence of coherent triplet yield on temperature

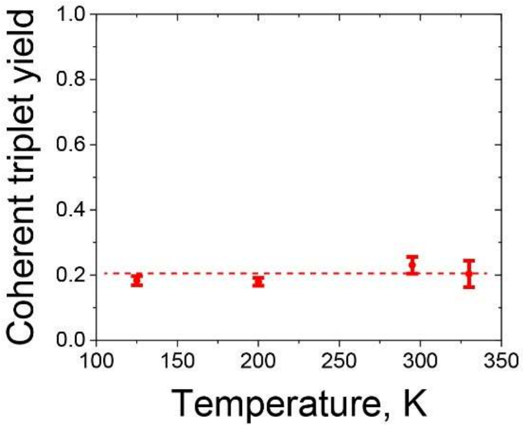

Fig. S17. Dependence of coherent triplet yield on temperature. The data from Figure 3 (main text) were used for evaluation.

Section XI. Computational methodology

The ground state (S_0) geometry of the rubrene molecule was optimized at the density functional theory (DFT) level using the B3LYP functional and 6-31G(d,p) basis set. Additionally, harmonic vibrational frequencies were calculated on the energy-minimized structure to ensure that the obtained geometry does not contain any imaginary frequencies. Subsequent time-dependent DFT (TD-DFT) calculations were carried out to optimize the geometries of the first singlet (S_1) and triplet (T_1) excited states. The Tamm-Dancoff approximation (TDA)[15] scheme was taken into account during the TDDFT calculations to avoid any instabilities related to triplet states. To determine the electron-vibrational couplings for the $S_0 \rightarrow T$ and $S_1 \rightarrow T$ transitions, we computed the Huang-Rhys (HR) factors between the relevant states. The computed HR factors are summarized in Tables S2-S4. All DFT calculations were performed with the Gaussian 16 package.[16] (We note that we initially aimed to utilize the long-range corrected ωB97X-D functional with an optimally tuned range-separation parameter to calculate the HR factors; however, we encountered technical issues that prevented us from getting reliable results.)

The S →TT transition rate constant (k) was estimated within the Marcus-Levich-Jortner[17] model:

$$k = \frac{2\pi}{\hbar} t^2 \times \frac{1}{\sqrt{4\pi\lambda_c k_B T}} \sum_{n=0}^{\infty} \frac{e^{-S_{qm}} \times S_{qm}^n}{n!} e^{\frac{-(\Delta E + \lambda_c + n\hbar\omega_{qm})^2}{4\lambda_c k_B T}} \quad \text{(S2)}$$

where t denotes electronic coupling; k_B, the Boltzmann constant; ħ, the reduced Plank constant; ΔE, the energy difference between S_0+S and T+T states; λ_c, the classical reorganization energy with low frequency modes (i.e., <100 cm-1); $\hbar\omega_{qm}$, the vibration energy of the effective high-frequency quantum mode; and Sqm, the Huang-Rhys factor associated with the reorganization energy from high-frequency modes.

We estimated ω_{qm} and S_{qm} as follows:

$$\omega_{qm} = \sqrt{\frac{(\sum \lambda_i \omega_i^2)_{S_0 \rightarrow T_1} + (\sum \lambda_i \omega_i^2)_{S_1 \rightarrow T_1}}{(\sum \lambda_i)_{S_0 \rightarrow T_1} + (\sum \lambda_i)_{S_1 \rightarrow T_1}}} \quad \text{(S3)}$$

$$\lambda_{qm} = \hbar\omega_{qm} S_{qm} \quad \text{(S4)}$$

where ω_i and λ_i are the frequency and the related reorganization energy of the vibrational mode *i*, while λ_{qm} denotes the reorganization energy due to high-frequency (quantum) vibrational modes. For the rate calculations, we considered the experimental temperature (T) range, from 125 K to 330 K. The S and T state energies were taken from reported experimental data as 2.23 eV and 1.14 eV, respectively, resulting in a ΔE value of 0.05 eV.[18] The low-frequency vibrational modes (see Tables S2-S4) with frequencies below 100 cm^{-1} were treated as classical, while the high-frequency modes (>100 cm^{-1}) were treated quantum mechanically. This leads to ω_{qm}=1368 cm^{-1}, λc=185 cm^{-1}, and S_{qm}=1.64. To take into account the medium effects and crystal lattice relaxations, we added an amount of 0.1 eV to λ_c.

In the 125 K – 330 K temperature range, the experimental rates of singlet fission (SF) show a variation from ~ 7×10^9 s^{-1} to 1.35×10^{11} s^{-1}, as illustrated in Figure 3f in the main text. In order to ensure that the computational SF rate derived in this study using the Marcus-Levich-Jortner equation (S2) aligns with the experimental values, it is crucial to select an appropriate electronic coupling in addition to the above-mentioned parameters. Therefore, we opted to use a small electronic coupling of either 60 cm^{-1} (when 0.1 eV is not added to λ_c) or 95 cm^{-1} (when 0.1 eV is added to λ_c), which ensures consistency of the computed rates with the experimental values.

Table S1 displays the SF rates calculated for different temperature ranges. By fitting the natural log (ln) of the SF rate vs $1/k_BT$, we obtain the activation energy (E_a) for $S_0S_1 \rightarrow T_1T_1$ to be 49.4 meV (Fig. S18). The inclusion of the environmental effects and crystal lattice relaxations to the classical reorganization energy (λ_c) increases E_a to 52.2 meV.

Table S1. SF rates at different temperatures as calculated using the Marcus-Levich-Jortner equation at the B3LYP/6-31G(d,p) level of theory. Rate-I corresponds to the case when 0.1 eV is not added to λc, while Rate-II corresponds to the case when 0.1 eV is added to λc.

T (K)	Rate-I (s^{-1})	Rate-II (s^{-1})
125	8.44×10^{9}	7.02×10^{9}
150	1.89×10^{10}	1.64×10^{10}
200	5.02×10^{10}	4.61×10^{10}
250	8.8×10^{10}	8.36×10^{10}
300	1.26×10^{11}	1.22×10^{11}
330	1.47×10^{11}	1.45×10^{11}

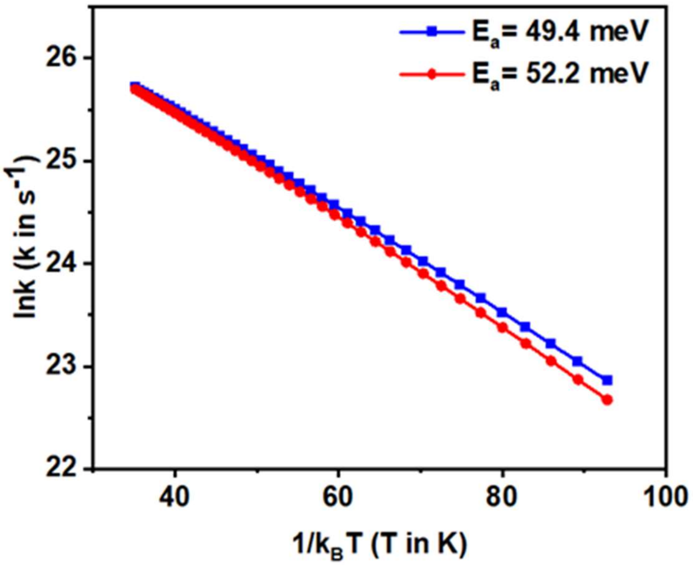

Fig. S18. Rate constants (k) as a function of inverse temperature (T) between 125 K and 330 K. The blue line is generated by adding 0.1 eV to λc to incorporate the effects of the medium and crystal lattice relaxations, while the red line does not consider such effects.

Table S2. Relaxation energies and Huang-Rhys (HR) factors related to the $S_0 \rightarrow S_1$ transition as computed at the B3LYP/6-31G(d,p) level of theory.

$S_0 \rightarrow S_1$			$S_1 \rightarrow S_0$		
Frequencies (ω in cm^{-1})	Relaxation energy (in cm^{-1})	(HR) factor	Frequencies (ω in cm^{-1})	Relaxation energy (in cm^{-1})	(HR) factor
23	249.00	10.826	27	354.20	13.119
69	42.80	0.620	67	6.30	0.094
80	27.60	0.345	76	67.30	0.886
134	0.20	0.001	133	0.70	0.005
214	82.60	0.386	218	1.60	0.007
253	1.60	0.006	249	30.90	0.124
265	4.10	0.015	264	1.20	0.005
339	53.10	0.157	338	81.10	0.240
416	1.80	0.004	416	4.80	0.012
450	5.30	0.012	432	2.20	0.005
535	4.30	0.008	526	9.10	0.017
624	3.80	0.006	616	50.70	0.082
631	16.90	0.027	627	35.30	0.056
659	0.60	0.001	643	0.10	0.000
674	270.10	0.401	670	36.40	0.054
718	2.10	0.003	717	2.10	0.003
767	0.30	0.000	757	2.80	0.004
781	0.50	0.001	770	0.70	0.001
858	1.70	0.002	857	0.00	0.000
909	149.10	0.164	910	415.30	0.456
924	28.80	0.031	924	33.70	0.036
969	0.10	0.000	939	22.30	0.024
977	4.40	0.005	972	1.40	0.001
993	14.00	0.014	992	1.10	0.001
1002	175.60	0.175	1014	220.10	0.217
1017	363.60	0.358	1015	248.80	0.245
1057	173.00	0.164	1058	180.80	0.171
1067	12.40	0.012	1088	0.80	0.001
1104	1.20	0.001	1107	2.70	0.002
1186	3.70	0.003	1186	3.50	0.003
1201	9.90	0.008	1197	73.50	0.061
1208	115.00	0.095	1208	110.80	0.092
1232	72.30	0.059	1243	54.10	0.044
1319	38.20	0.029	1319	4.60	0.003
1347	234.40	0.174	1354	8.10	0.006
1353	14.20	0.010	1360	1.50	0.001

1357	27.10	0.020	1372	224.60	0.164
1477	53.00	0.036	1423	196.80	0.138
1482	8.70	0.006	1481	7.90	0.005
1529	14.50	0.009	1530	38.80	0.025
1542	10.50	0.007	1540	93.00	0.060
1592	236.80	0.149	1590	175.40	0.110
1629	3.20	0.002	1624	3.90	0.002
1656	5.80	0.004	1649	21.60	0.013
3174	14.60	0.005	3175	21.80	0.007
3182	0.40	0.000	3182	0.90	0.000
3192	66.50	0.021	3194	49.00	0.015
3197	0.00	0.000	3200	10.90	0.003
3199	14.50	0.005	3201	0.30	0.000
3207	269.60	0.084	3207	280.60	0.087
3240	1.40	0.000	3238	1.30	0.000
Total relaxation energy					
	0.361 eV			0.397 eV	

Table S3. Relaxation energies and Huang-Rhys (HR) factors related to the $S_0 \rightarrow T_1$ transition as computed at the B3LYP/6-31G(d,p) level of theory.

$S_0 \rightarrow T_1$			$T_1 \rightarrow S_0$		
Frequencies (ω in cm^{-1})	Relaxation energy (in cm^{-1})	(HR) $_{S0 \rightarrow T1}$	Frequencies (ω in cm^{-1})	Relaxation energy (in cm^{-1})	(HR) $_{T1 \rightarrow S0}$
23	101.00	4.391	23	104.30	4.535
69	6.00	0.087	66	0.10	0.002
80	5.90	0.074	78	8.30	0.106
134	22.50	0.168	128	17.70	0.138
214	31.70	0.148	215	6.50	0.030
253	4.70	0.019	250	17.80	0.071
265	12.30	0.046	266	10.40	0.039
339	83.40	0.246	335	108.70	0.324
416	0.00	0.000	415	0.00	0.000
450	16.90	0.038	437	5.50	0.013
535	6.80	0.013	529	10.50	0.020
624	0.30	0.000	609	6.10	0.010
631	0.40	0.001	630	19.50	0.031
659	1.20	0.002	641	2.20	0.003
674	70.10	0.104	673	2.20	0.003
718	0.30	0.000	717	0.20	0.000
767	1.60	0.002	765	7.20	0.009
781	7.50	0.010	772	1.00	0.001
858	0.20	0.000	858	0.10	0.000
909	0.30	0.000	912	82.20	0.090
924	1.60	0.002	925	4.60	0.005
969	0.40	0.000	949	12.60	0.013
977	4.60	0.005	972	0.60	0.001
993	11.30	0.011	993	0.00	0.000
1002	138.80	0.139	1015	15.40	0.015
1017	28.10	0.028	1018	42.20	0.041
1057	14.10	0.013	1058	19.90	0.019
1067	2.20	0.002	1094	0.80	0.001
1104	0.10	0.000	1106	3.90	0.004
1186	0.40	0.000	1186	0.50	0.000
1201	54.40	0.045	1196	121.80	0.102
1208	11.60	0.010	1208	9.20	0.008

1232	7.30	0.006	1238	1.90	0.002
1319	68.80	0.052	1303	259.50	0.199
1347	488.90	0.363	1324	32.50	0.025
1353	94.60	0.070	1357	6.30	0.005
1357	91.40	0.067	1367	395.60	0.289
1477	22.20	0.015	1405	185.40	0.132
1482	0.30	0.000	1480	6.00	0.004
1529	103.70	0.068	1529	140.10	0.092
1542	52.50	0.034	1540	115.00	0.075
1592	402.20	0.253	1611	195.80	0.122
1629	0.50	0.000	1627	0.00	0.000
1656	0.40	0.000	1654	2.80	0.002
3174	1.30	0.000	3175	1.60	0.001
3182	0.00	0.000	3183	0.10	0.000
3192	6.60	0.002	3193	5.40	0.002
3197	0.20	0.000	3199	0.70	0.000
3199	1.80	0.001	3200	0.80	0.000
3207	27.70	0.009	3207	28.00	0.009
3240	0.10	0.000	3235	0.40	0.000
Total relaxation energy					
	0.249 eV			0.251	

Table S4. Relaxation energies and Huang-Rhys (HR) factors related to the $S_1 \rightarrow T_1$ transition as computed at the B3LYP/6-31G(d,p) level of theory.

$S_1 \rightarrow T_1$			$T_1 \rightarrow S_1$		
Frequencies (ω in cm^{-1})	Relaxation energy (in cm^{-1})	(HR) $_{S1 \rightarrow T1}$	Frequencies (ω in cm^{-1})	Relaxation energy (in cm^{-1})	(HR) $_{T1 \rightarrow S1}$
27	47.30	1.752	23	34.70	1.509
67	4.80	0.072	66	6.30	0.095
76	19.90	0.262	78	12.80	0.164
133	16.70	0.126	128	17.30	0.135
218	0.00	0.000	215	1.70	0.008
249	0.00	0.000	250	0.20	0.001
264	2.90	0.011	266	2.20	0.008
338	3.70	0.011	335	4.20	0.013
416	4.20	0.010	415	2.30	0.006
432	7.90	0.018	437	5.50	0.013
526	0.20	0.000	529	0.60	0.001
616	2.70	0.004	609	1.60	0.003
627	0.10	0.000	630	5.60	0.009
643	1.90	0.003	641	4.10	0.006
670	3.30	0.005	673	5.30	0.008
717	0.10	0.000	717	0.10	0.000
757	1.30	0.002	765	0.60	0.001
770	1.00	0.001	772	1.20	0.002
857	0.20	0.000	858	0.50	0.001
910	4.20	0.005	912	18.30	0.020
924	1.50	0.002	925	0.40	0.000
939	0.00	0.000	949	0.70	0.001
972	0.00	0.000	972	0.00	0.000
992	0.40	0.000	993	0.10	0.000
1014	47.60	0.047	1015	2.60	0.003
1015	1.70	0.002	1018	19.10	0.019
1058	6.00	0.006	1058	8.50	0.008
1088	0.00	0.000	1094	0.00	0.000
1107	0.80	0.001	1106	1.30	0.001
1186	0.00	0.000	1186	0.10	0.000
1197	3.80	0.003	1196	6.60	0.006
1208	5.70	0.005	1208	4.50	0.004

1243	6.80	0.005	1238	0.40	0.000
1319	10.70	0.008	1303	98.70	0.076
1354	34.50	0.025	1324	18.40	0.014
1360	46.00	0.034	1357	0.10	0.000
1372	45.20	0.033	1367	10.20	0.007
1423	0.20	0.000	1405	7.30	0.005
1481	0.30	0.000	1480	0.60	0.000
1530	18.00	0.012	1529	21.80	0.014
1540	35.40	0.023	1540	11.30	0.007
1590	16.40	0.010	1611	12.50	0.008
1624	0.10	0.000	1627	0.00	0.000
1649	2.20	0.001	1654	0.00	0.000
3175	1.00	0.000	3175	0.60	0.000
3182	0.00	0.000	3183	0.00	0.000
3194	1.80	0.001	3193	2.30	0.001
3200	0.40	0.000	3199	0.20	0.000
3201	0.00	0.000	3200	0.20	0.000
3207	10.20	0.003	3207	9.90	0.003
3238	0.00	0.000	3235	0.20	0.000
Total relaxation energy					
	0.052 eV			0.045 eV	

Vibronic model

In order to simulate concomitantly the absorption spectrum of the rubrene molecule and to account for the **S:TT** coupling, we used a 3-state model comprising the ground state and the S_1 and ^1TT states:

$$H_{vib} = \begin{pmatrix} E_G + H_{vib}^{(G)} & -(\vec{d}\vec{E}) & 0 \\ -(\vec{d}\vec{E}) & E_S + H_{vib}^{(S)} & t \\ 0 & t & E_{TT} + H_{vib}^{(TT)} \end{pmatrix} \quad \text{(S5)}$$

$$H_{vib}^{(G)} = \sum_i \frac{\hbar\omega_i}{2} q_i^2 \quad \text{(S6)}$$

$$H_{vib}^{(S)} = \sum_i \frac{\hbar\omega_i}{2} q_i^2 + \sum_i \sqrt{2}\ \hbar\omega_i g_i^{(S)} + \lambda_s \quad \text{(S7)}$$

$$H_{vib}^{(TT)} = \sum_i \frac{\hbar\omega_i}{2} q_i^2 + \sum_i \sqrt{2}\ \hbar\omega_i g_i^{(TT)} + \lambda_{TT} \quad \text{(S8)}$$

$$\lambda = \sum_i \hbar\omega_i\ g_i^2 \quad \text{(S9)}$$

Here, E_G, E_S *and* E_{TT} are, respectively, the energies of the ground state and the S and TT states; ***t*** is the coupling between the S and TT states; ω_i and g_i are, respectively, the frequency and the coupling constant of the vibrational mode q_i. $H_i = -(\vec{d}\vec{E})$ describes the coupling of the S_0 to S electronic excitation with the applied electromagnetic field (light) in the dipole approximation. This interaction is treated here perturbatively.

The dynamic solutions (Eq. S5) of the vibronic Hamiltonian were obtained numerically using a procedure described in our previous work.[19] Since the H_i was treated as a perturbation, the vibronic eigenfunctions accounts only for the hybridization between S and TT states and are given as:

$$\psi_\alpha = \chi_\alpha^{(S)}(q)\psi^{(S)} + \chi_\alpha^{(TT)}(q)\psi^{(TT)} \quad \text{(S10)}$$

Here, $\psi^{(M)}$ denotes the electronic wavefunction of state M and $\chi_\alpha^{(M)}(q)$ is the related vibrational function where q stands for the normal coordinates of all involved vibrational modes. The dynamic solutions of the vibronic Hamiltonian can be obtained by expending the functions

$\chi_{\alpha}^{(M)}(q)$ in terms of harmonic-oscillator eigen-functions. By using a large but finite number of these functions in the expansion, one can obtain the vibronic solutions with any desirable accuracy. We note that the coefficients $\left|\chi_{\alpha}^{(S)}\right|^2$ and $\left|\chi_{\alpha}^{(S)}\right|^2$ obtained after integration over q satisfy the standard normalization condition ($\left|\chi_{\alpha}^{(S)}\right|^2 + \left|\chi_{\alpha}^{(S)}\right|^2 = 1$) and can be taken as measure of S and TT contributions to the hybrid **S**:**TT** states**.**

If only the transitions from the ground vibronic state are considered, the absorption spectrum is proportional to $P_{0\alpha}^{(S)} = \left|\chi_{0}^{(G)}(q)\,\chi_{\alpha}^{(G)}(q)\right|^2$. The weight of the **TT** state to vibronic state α is given by $P_{\alpha}^{(TT)} = \left|\chi_{\alpha}^{(TT)}(q)\,\chi_{\alpha}^{(TT)}(q)\right|^2$. As a consequence, the product $P_{0\alpha} * P_{\alpha}^{(TT)}$ gives the probability of generating a TT state by means of photoexcitation. The probability, $(P^{(S)} * P^{(TT)})(E)$, of generating a TT state with energy E, is obtained by broadening the vibronic transition energies (using Gaussian functions) and summing up $P_{0\alpha} * P_{\alpha}^{(TT)}$ over all transitions. Finally, the probability of generating a TT state per absorbed photon with energy E, (referred here as the TT photogeneration population) was computed by dividing $(P^{(S)} * P^{(TT)})(E)$ by $P^{(S)}(E)$.

In the vibronic calculations, we used two effective vibrational modes, one high-frequency mode (ω_1) and one low-frequency vibrational mode (ω_2). The parameters in the $H_{vib}^{(S)}$ term (Eq. S7) were chosen such that the calculations reproduce the experimental absorption spectrum of isolated rubrene molecules. The coupling to a low energy (ω_2=200 cm^{-1}) vibrational mode was considered only in the $H_{vib}^{(TT)}$ term (Eq. S8). The vibrational constants entering $H_{vib}^{(TT)}$ were chosen to reproduce the reorganization energy related to **S** →**TT** transition.

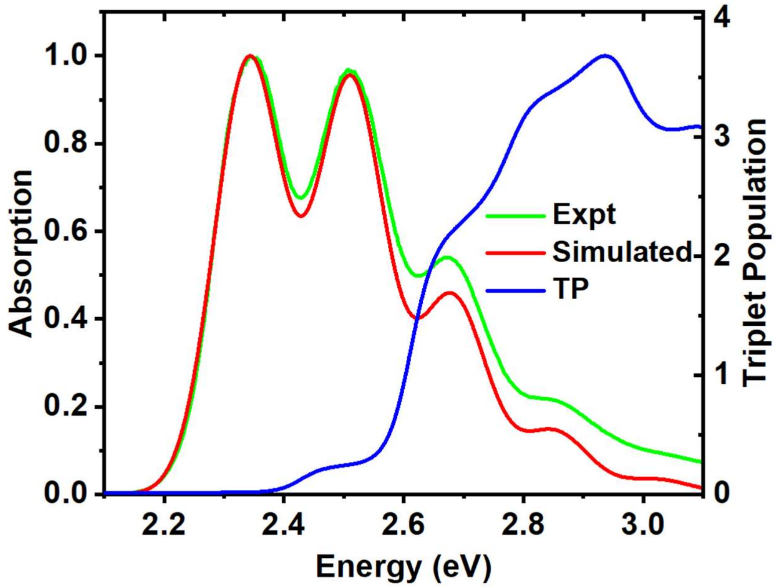

Fig. S19. S1 absorption of rubrene in deuterated chloroform solution (green curve), its simulation (red curve) and the relative weight (population) of the **TT** *state normalised per absorbed photon at a given energy (blue curve). The vibronic Hamiltonian was solved by using the following microscopic parameters: t = 7.4 meV , ω_1=1370 cm^{-1} , ω_2=200 cm^{-1}, $g_1^{(S)}$= -0.975, $g_2^{(S)}$ = 0, $g_1^{(TT)}$ = 0.1, and $g_2^{(TT)}$ = 4.*

Supplementary References:

1. Podzorov, V. Organic single crystals: Addressing the fundamentals of organic electronics. *MRS Bull.* **38**, (2013).

2. de Boer, R. W. I., Gershenson, M. E., Morpurgo, A. F. & Podzorov, V. Organic single-crystal field-effect transistors. *Phys. status solidi* **201**, 1302–1331 (2004).

3. Bruevich, V., Choi, H. H. & Podzorov, V. The Photo-Hall Effect in High-Mobility Organic Semiconductors. *Adv. Funct. Mater.* **31**, 2006178 (2021).

4. Cruz, C. D., Chronister, E. L. & Bardeen, C. J. Using temperature dependent fluorescence to evaluate singlet fission pathways in tetracene single crystals. *J. Chem. Phys.* **153**, 234504 (2020).

5. Chen, Y., Lee, B., Fu, D. & Podzorov, V. The Origin of a 650 nm Photoluminescence Band in Rubrene. *Adv. Mater.* **23**, 5370–5375 (2011).

6. Lyons, L. E. & Warren, L. J. Anthracene fluorescence at low temperatures. II. Doped single crystals. *Aust. J. Chem.* **25**, 1427–1441 (1972).

7. Parashchuk, O. D. *et al.* Molecular Self-Doping Controls Luminescence of Pure Organic Single Crystals. *Adv. Funct. Mater.* **28**, 1800116 (2018).

8. Yi, H. T., Gartstein, Y. N. & Podzorov, V. Charge carrier coherence and Hall effect in organic semiconductors. *Sci. Rep.* **6**, 23650 (2016).

9. Choi, H. H. *et al.* Accurate Extraction of Charge Carrier Mobility in 4-Probe Field-Effect Transistors. *Adv. Funct. Mater.* **28**, 1707105 (2018).

10. Chen, Y., Yi, H. T. & Podzorov, V. High-Resolution ac Measurements of the Hall Effect in Organic Field-Effect Transistors. *Phys. Rev. Appl.* **5**, 034008 (2016).

11. Maimaris, M. *et al.* Sub-10-fs observation of bound exciton formation in organic optoelectronic devices. *Nat. Commun.* **13**, 4949 (2022).

12. Ning, H. *et al.* Ultrafast Broadband Strong-Field Tunnelling in Asymmetric Nanogaps for

Time-Resolved Nanoscopy (2024).

13. Materials-Studio-Modeling, Accelrys Software Inc (2009).

14. Sun, H. COMPASS: An ab Initio Force-Field Optimized for Condensed-Phase ApplicationsOverview with Details on Alkane and Benzene Compounds. *J. Phys. Chem. B* **102**, 7338–7364 (1998).

15. Hirata, S. & Head-Gordon, M. Time-dependent density functional theory within the Tamm–Dancoff approximation. *Chem. Phys. Lett.* **314**, 291–299 (1999).

16. Frisch, M. J. *et al.* G16_C01. Gaussian 16, Revision C.01, Gaussian, Inc., Wallin at (2016).

17. Chen, P. & Meyer, T. J. Medium Effects on Charge Transfer in Metal Complexes. *Chem. Rev.* **98**, 1439–1478 (1998).

18. Pandey, A. K. Highly efficient spin-conversion effect leading to energy up-converted electroluminescence in singlet fission photovoltaics. *Sci. Rep.* **5**, 7787 (2015).

19. Chen, X.-K., Coropceanu, V. & Brédas, J.-L. Assessing the nature of the charge-transfer electronic states in organic solar cells. *Nat. Commun.* **9**, 5295 (2018).
